# CTCF-mediated chromatin looping in EGR2 regulation and SUZ12 recruitment critical for peripheral myelination and repair

**DOI:** 10.1038/s41467-020-17955-2

**Published:** 2020-08-17

**Authors:** Jincheng Wang, Jiajia Wang, Lijun Yang, Chuntao Zhao, Laiman Natalie Wu, Lingli Xu, Feng Zhang, Qinjie Weng, Michael Wegner, Q. Richard Lu

**Affiliations:** 1 Center for Drug Safety Evaluation and Research, College of Pharmaceutical Sciences, Zhejiang University, Hangzhou, 310058 China; 2 Department of Pediatrics, Brain Tumor Center, Division of Experimental Hematology and Cancer Biology, Cincinnati Children’s Hospital Medical Center, Cincinnati, OH 45229 USA; 3 Institut für Biochemie, Emil-Fischer-Zentrum, Friedrich-Alexander-Universität Erlangen-Nürnberg, Erlangen, Germany

**Keywords:** Developmental neurogenesis, Chromatin structure, Development of the nervous system, Epigenetics in the nervous system, Glial biology

## Abstract

Chromatin organization is critical for cell growth, differentiation, and disease development, however, its functions in peripheral myelination and myelin repair remain elusive. In this report, we demonstrate that the CCCTC-binding factor (CTCF), a crucial chromatin organizer, is essential for Schwann cell myelination and myelin regeneration after nerve injury. Inhibition of CTCF or its deletion blocks Schwann cell differentiation at the pro-myelinating stage, whereas overexpression of CTCF promotes the myelination program. We find that CTCF establishes chromatin interaction loops between enhancer and promoter regulatory elements and promotes expression of a key pro-myelinogenic factor EGR2. In addition, CTCF interacts with SUZ12, a component of polycomb-repressive-complex 2 (PRC2), to repress the transcriptional program associated with negative regulation of Schwann cell maturation. Together, our findings reveal a dual role of CTCF-dependent chromatin organization in promoting myelinogenic programs and recruiting chromatin-repressive complexes to block Schwann cell differentiation inhibitors to control peripheral myelination and repair.

## Abstract (web summary)

Myelination by Schwann cells (SC) in the peripheral nervous system is essential for motor function, and dysregulation of SC myelination can lead to various neuropathies. Here the authors describe a critical role of CCCTC-binding factor (CTCF)-dependent chromatin reorganization in peripheral myelination and myelin regeneration after injury.

## Introduction

High-order chromatin organization and remodeling are critical for fundamental biological processes^1–3^. Local chromatin environments that modulate recruitment of transcriptional complexes to regulatory elements are highly dynamic and depend on stage- or cell-type-specific nucleosome positions or chromatin looping^1–3^. The chromatin reorganization process enables long-range interactions such as those between promoters and enhancers that activate gene transcription. In addition, insulator-mediated contacts can organize the genome into functionally distinct domains^1–3^. Defects in chromatin structural organization or looping can cause aberrant transcriptional regulation, leading to various diseases including intellectual disabilities, cancer, and aging^4–6^.

Schwann cells (SCs) are myelinating glia in the peripheral nervous system (PNS) that form myelin sheaths around axons to optimize the saltatory nerve conduction. Defects in SCs lead to various peripheral neuropathies including motor and sensory disabilities^7,8^. SC-lineage development includes the specification of neural crest cells to SC precursors that give rise to immature SCs, which further differentiate into mature myelinating SCs^9^. The process of SC development is regulated by various intrinsic and extrinsic cues. Among intrinsic factors, transcriptional regulators such as SOX10, OCT6 (a.k.a. POU3F1), and EGR2 (a.k.a. KROX20) are required for sequential progression from immature to promyelinating SCs, and eventually into myelinating SCs^10–12^. SC development is coordinated by a hierarchy of transcriptional regulators with a main axis that SOX10 activates OCT6, and then cooperates with OCT6 to induce EGR2 expression for SC maturation^11–13^. EGR2 takes a center stage for myelinogenesis by activating myelin genes, such as *Mpz*, *Pmp22*, and *Mbp*^14,15^. The negative regulatory cues that inhibit SC myelination include NOTCH, WNT, and SOX2 pathways^12,16,17^. How chromatin reorganizes to promote expression of promyelin cues while preventing the differentiation-inhibitory events during SC myelination has not been determined.

CCCTC-binding factor (CTCF) is one of the most critical organizers for the high-order chromatin structure that enables long-range chromatin interactions^3,18,19^. Accumulating evidence suggests that CTCF mediates extensive crosstalk between promoters and distant regulatory elements and regulates local balance between active and repressive chromatin marks, therefore ensuring proper transcription levels during various biological processes^3,18,19^. CTCF can not only mediate long-range chromatin looping and modulate three-dimensional genomic architecture to regulate cell-type-specific transcriptional programs^20–22^, but also define boundaries between chromosomal topological associating domains (TADs)^23,24^. To date, the function of CTCF-dependent long-range chromatin interactions and looping in peripheral myelination and regeneration has not been defined. In addition, given its ubiquitous role in gene regulation by CTCF, whether CTCF has a temporally specific role during SC myelination remains elusive.

The specific local genomic architecture also depends on histone modifications, DNA modification patterns, and nucleosome positioning or accessibility to maintain the proper conformation for transcription^25^. Histone modifying enzymes such as the deacetylases HDAC1/2 and polycomb-repressive complex 2 (PRC2) modulate chromatin states to regulate the transcriptional program necessary for myelination and remyelination in the PNS^26–28^. At present, however, it is unknown how chromatin dynamics coordinate histone modifications to control SC myelination programs.

Here, we demonstrate a critical role of CTCF-dependent chromatin reorganization during SC differentiation from their immature precursors and in remyelination after peripheral nerve injury. We show that CTCF interacts with and recruits SUZ12 to suppress SC differentiation-inhibitory pathways. Furthermore, we find that CTCF regulates promyelination transcriptional programs at least in part by establishing an interaction between promoter and enhancer elements of the locus of *Egr2*, a key regulatory gene for SC myelination^12^. Thus, our data demonstrate a temporally specific function of the chromatin organizer CTCF for SC differentiation by modulating chromatin organization and epigenetic programs to control peripheral myelination.

## Results

### Upregulation of CTCF expression during SC differentiation

**Fig. 1:**
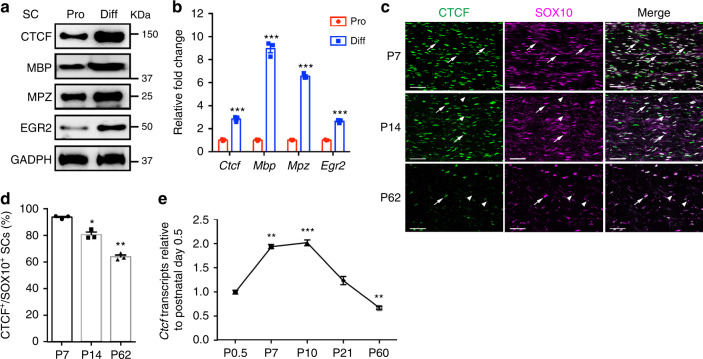
CTCF expression changes during SC-lineage progression. **a** Western blots for CTCF, MBP, MPZ, and EGR2 in proliferating and differentiated rat SC cultures. GAPDH served as a loading control. *n* = 2 independent experiments. **b** Relative qPCR expression of *Ctcf*, *Mbp*, *Mpz*, and *Egr2* in proliferating and differentiated rat SC cultures. Data are presented as means ± SEM., ***P < 0.001, *n* = 3 independent experiments; two-tailed unpaired Student’s *t*-test, *P*_(*Ctcf)*_ = 0.00021, *P*_(*Mbp)*_ = 2.8E-05, *P*_(*Mpz)*_ = 1.7E-06, *P*_(*Egr2)*_ = 3.9E-05. **c** Colocalization of CTCF with SOX10 in SC nuclei from mice at P7, P14, and P62 evaluated by immunofluorescence labeling. Representative images are shown. *n* = 3 nerve tissues at each time point. Arrows indicate SOX10^+^/CTCF^+^ SCs; arrowheads indicate SOX10^+^/CTCF^−^ SCs. Scale bars: 50 μm. **d** The percentage of CTCF^+^ nuclei in SCs (SOX10^+^) in sciatic nerves from P7, P14, and P62 mice. *n* = 3 control tissues at each time point. Data are presented as means ± SEM., *P < 0.05, **P < 0.01; *n* = 3 nerve tissues at each time point; one-way ANOVA with multiple comparisons test. *P*_(P14)_ = 0.0392, *P*_(P62)_ = 0.0052. **e** Relative qPCR expression of *Ctcf* in mouse sciatic nerves at various developmental stages. Data are presented as means ± SEM., **P < 0.01, ***P < 0.001; *n* = 3 nerve tissues at each time point; one-way ANOVA with multiple comparisons test, *P*_(P7)_ = 0.0067, *P*_(P10)_ = 0.0004, *P*_(P21)_ = 0.1503, *P*_(P60)_ = 0.0077. Source data are provided as a Source Data file.

To investigate the expression pattern of CTCF in proliferative and differentiated SCs, we treated rat SCs with cAMP to promote their differentiation in vitro. Expression of mature SC markers such as EGR2, MBP, and MPZ increased during differentiation. Strikingly, CTCF protein and mRNA expression levels were also elevated during SC differentiation (Fig. 1a, b).

To characterize CTCF expression in vivo, we co-immunostained CTCF with SC-lineage marker SOX10 in sciatic nerves (Fig. 1c). At postnatal day (P) 7, the majority of the SCs marked by SOX10 were also CTCF-positive. CTCF expression persisted in SCs in late developmental stages, but decreased in adulthood (Fig. 1c, d). To confirm these changes, we performed qRT-PCR analysis of mouse sciatic nerves at different stages and found that *Ctcf* transcripts were detected at the neonatal stage P0.5, an immature SC stage. *Ctcf* levels peaked at the perinatal stage P10, when the majority of SCs undergo differentiation and then were gradually reduced in adulthood (Fig. 1e).

### CTCF is critical for SC differentiation

**Fig. 2:**
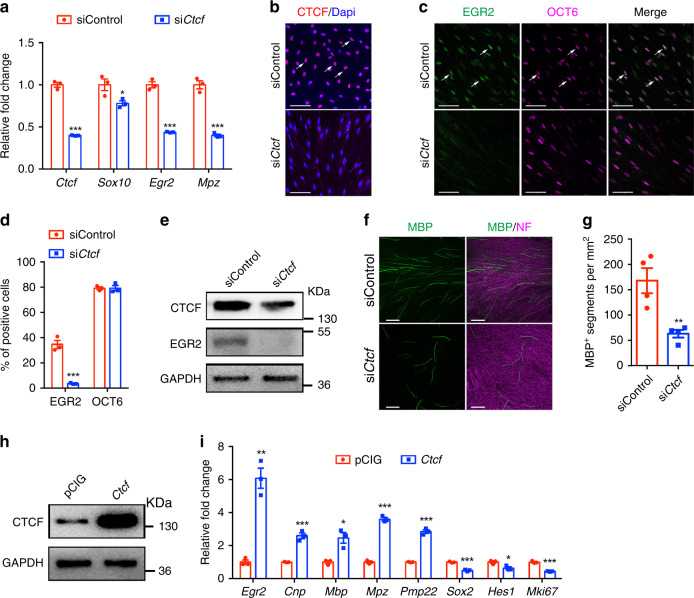
CTCF is critical for rat SC differentiation in vitro. **a** qRT-PCR analysis of *Ctcf*, *Sox10*, *Egr2*, and *Mpz* expression in rat SCs transfected with control nontargeting siRNA and si*Ctcf* for 24 h and induced to differentiate for 9 h. *n* = 3 independent experiments, *P*_(*Ctcf*)_ = 3.03E-05, *P*_(*Sox10*)_ = 0.0433, *P*_(*Egr2*)_ = 0.000107, *P*_(*Mpz*)_ = 0.000293. **b–d** Rat SCs were transfected with control siRNA or si*Ctcf* for 24 h and induced to differentiate for 9 h and CTCF- (**b**), EGR2- and OCT6-positive (**c**) cells were visualized by immunofluorescence microscopy and **d** quantified; *n* = 3 independent experiments. Arrows indicate CTCF^+^ or EGR2^+^/OCT6^+^ SCs. Scale bars: 50 µm. *n* = 3 independent experiments, *P*_(EGR2)_ = 0.00069, *P*_(OCT6)_ = 0.99. **e** Western blots for CTCF and EGR2 in co-cultures of rat DRGs and SCs treated with control siRNA or si*Ctcf*. GAPDH served as a loading control. *n* = 4 independent experiments. **f** Rat SCs treated with control siRNA or si*Ctcf* were seeded onto rat DRGs. After 10 days, co-cultures were immunostained for MBP and neurofilament-M. Images are representative of *n* = 4 independent experiments. Scale bars: 100 μm. **g** Quantification of the number of MBP^+^ segments per mm^2^ of area in myelinating co-cultures of DRGs and SCs treated with control siRNA or si*Ctcf*. *n* = 4 independent experiments, *P* = 0.0068. **h** Western blots for CTCF in rat Schwann cells induced to differentiate following transfection with control or CTCF expression vectors. *n* = 2 independent experiments. **i** qRT-PCR quantification of differentiation regulators and negative regulators in rat SCs induced to differentiate following transfection with control or CTCF expression vectors. *n* = 3 independent experiments, *P*_(*Egr2*)_ = 0.0012, *P*_(*Cnp*)_ = 0.00068, *P*_(*Mbp*)_ = 0.011, *P*_(*Mpz*)_ = 2.9E-05, *P*_(*Pmp22*)_ = 6.7E-05, *P*_(*Sox2*)_ = 0.00026, *P*_(*Hes1*)_ = 0.028, *P*_(*Mki67*)_ = 0.00024. Data are presented as means ± SEM., *P < 0.05, **P < 0.01, ***P < 0.001, two-tailed unpaired Student’s *t*-test. Source data are provided as a Source Data file.

To investigate the functions of CTCF in SC differentiation, we transfected cultured rat SCs with a siRNA designed to target CTCF. Levels of pro-SC differentiation genes, *Sox10, Egr2*, and a SC myelin gene *Mpz*, were significantly reduced in differentiated SCs deficient in CTCF (Fig. 2a). Similarly, expression of the promyelination factor EGR2 was diminished in SCs treated with si*Ctcf* (Fig. 2b–d). In contrast, expression of OCT6, which marks promyelinating SCs, was not significantly altered (Fig. 2c, d). These results suggest that CTCF deficiency blocks SC differentiation beyond the promyelinating stage.

To further determine the function of CTCF in myelination, we silenced *Ctcf* in ex vivo rat SC-dorsal root ganglion co-cultures. We observed a strong reduction of MBP^+^ myelin formation along the axons (Fig. 2e–g). In contrast, *Ctcf* overexpression elevated *Egr2* and myelination-associated genes (e.g., *Mbp*, *Mpz*, and *Pmp22*) and repressed expression of immature SC and proliferation-associated genes, such as *Sox2*, *Hes1*, and *Mki67* (Fig. 2h, i), consistent with that CTCF overexpression blocks cell-cycle progression in a variety of cell lines^29^. Thus, our data suggests that CTCF is necessary to promote the SC differentiation program.

### Mice lacking CTCF display severe peripheral hypomyelination

**Fig. 3:**
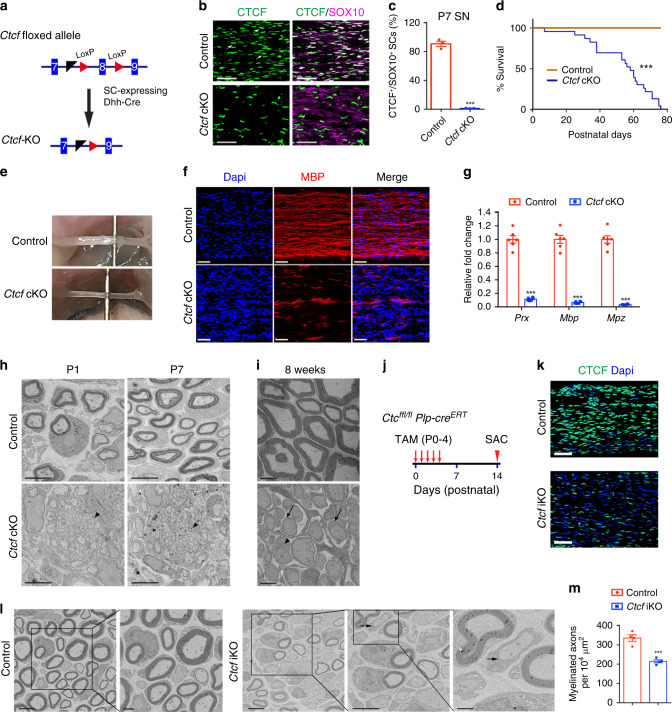
CTCF is required for peripheral nerve ensheathment. **a** Excised exon 8 of the floxed *Ctcf* allele by *Dhh-Cre*. **b** Co-labeling of CTCF with SOX10 in control and mutant sciatic nerves at P7 (*n* = 3 animals/genotype). Arrows indicate SOX10^+^/CTCF^+^ SCs. Scale bars: 50 μm. **c** The percentage of CTCF^+^ nuclei in SCs (SOX10^+^) from control and *Ctcf* cKO sciatic nerves at P7. *n* = 3 animals/genotype, *P* = 1.73E-05. **d** Survival curves of control and *Ctcf* cKO mice. *n* = 25 for control and *n* = 23 for *Ctcf* cKO mice, ***P < 0.001. **e** Representative photographs of sciatic nerves from P13 control and *Ctcf* cKO mice. *n* = 3 animals/genotype. **f** Immunofluorescence labeling of MBP (red) in P7 control and *Ctcf* cKO sciatic nerves. *n* = 3 animals/genotype. Scale bars: 50 μm. **g** The mRNA levels of myelin-related genes in P7 control and *Ctcf* cKO sciatic nerves. *n* = 6 animals/genotype. *P*_(*Prx*)_ = 1.9E-08, *P*_(*Mbp*)_ = 2.0E-08, *P*_(*Mpz*)_ = 8.5E-09. **h, i** Ultrastructure of control and *Ctcf* cKO sciatic nerves at (**h**) P1 and P7 and at (**i**) 8 weeks. *n* = 3 animals/genotype. Arrows and arrowheads indicate immature SCs and unsorted axons, respectively. Scale bars: 4 μm. **j** A diagram showing the tamoxifen (TAM) administration scheme. **k** Immunofluorescent labeling of CTCF (green) nuclei in control and *Ctcf* iKO sciatic nerves at P14. Scale bars: 50 μm. *n* = 3 animals/genotype. **l** EM images of P14 sciatic nerves from control and *Ctcf* iKO mice. *n* = 4 animals/genotype. Arrow indicates myelin membrane. Scale bars: 4 μm, and 1 μm in the inset on the right panel. **m** Myelinated axon numbers 10^−4^ μm^−2^ sections of P14 sciatic nerves from control and *Ctcf* iKO mice. *n* = 4 animals/genotype, *P* = 0.0006. Data are presented as means ± SEM., ***P < 0.001; Statistical analyses performed using two-tailed unpaired Student’s *t*-test; Log-rank test used for survival curve. Source data are provided as a Source Data file.

To investigate the SC-specific function of CTCF, we bred the *Ctcf* floxed mice (*Ctcf*^*loxP*/*loxP*^) with SC-lineage expressing *Desert hedgehog* (*Dhh*)-*Cre* line^10^ to ablate CTCF (Fig. 3a–c). *Ctcf*^*loxP/loxP*^*;Dhh-Cre* (referred as *Ctcf* cKO) mice appeared normal compared with littermate controls during the first postnatal week. Starting in the second week, however, all *Ctcf* cKO mice developed an unsteady gait and hindlimb paralysis, and the majority died by P80 (Fig. 3d). At P13, *Ctcf* cKO sciatic nerves had a thin and translucent appearance, in contrast to the thick, opaque nerves of control mice, suggesting a severe deficit in myelination (Fig. 3e).

To assess myelinogenesis in *Ctcf* cKO mice, we immunostained for MBP in P7 peripheral nerves. Compared with the robust expression in control mice, sparse expression of MBP in sciatic nerves of mutants suggested severe hypomyelination (Fig. 3f). Accordingly, expression of the myelin-associated genes, *Prx*, *Mbp*, and *Mpz*, was substantially decreased in mutant nerves (Fig. 3g). Electron microscopy (EM) revealed that in control mice at P1 and P7, SCs were present in a 1:1 relationship with large-caliber axons and the myelin membrane had begun wrapping around axons (Fig. 3h). In contrast, in *Ctcf* cKO mice, the majority of SCs lacked peripheral myelination and showed abnormal axon bundles, and most SCs failed to establish 1:1 relationships with axons (Fig. 3h). There was a partial radial sorting defect at neonatal stages (Fig. 3h, arrowheads), which persists into adulthood. Among the few mutant mice that survived to adulthood (Fig. 3d), the gross structure of *Ctcf* cKO peripheral nerves at P56 revealed no apparent sign of myelination by SCs (Fig. 3i). These observations suggest that *Ctcf* loss results in both a radial sorting defect and a failure of promyelinating SCs to proceed to myelination in peripheral nerves.

### CTCF deletion in postnatal immature SCs blocks myelination

Since Dhh-Cre inactivated *Ctcf* in early immature SCs, we then evaluated the impact of CTCF loss in SC myelination during postnatal development by deleting *Ctcf* in promyelinating SCs in neonatal peripheral nerves by using a tamoxifen-inducible *Plp-CreERT* driver^30^. Tamoxifen administration from P0 to P4 led to efficient downregulation of CTCF in the sciatic nerves of *Ctcf*^*loxP/loxP*^*;Plp-creERT2* mice (*Ctcf* iKO; Fig. 3j, k). EM analysis indicated that ~36% of large axons remained unmyelinated in mutant nerves (Fig. 3l, m), suggesting that CTCF is required for proper initiation of SC myelinogenesis. We detected a very thin layer of myelin sheath in some nonmyelinated large diameter axons (Fig. 3l, arrow), suggesting that a thin layer of myelin membrane could be formed before myelination arrest in *Ctcf* iKO mice.

The reduced expression of CTCF in mature nerves (Fig. 1f–h) suggested a nonessential function in myelin maintenance. Consistent with this hypothesis, neither the myelin sheath thickness nor its integrity was affected in peripheral nerves of adult mice in which tamoxifen-induced deletion of *Ctcf* was carried out at 4–6 weeks of age (Supplementary Fig. 1). Thus, our data indicate a central role of CTCF in initiating SC myelination but not in maintaining myelin sheath assembly.

### CTCF is required for SC myelinogenic programs

**Fig. 4:**
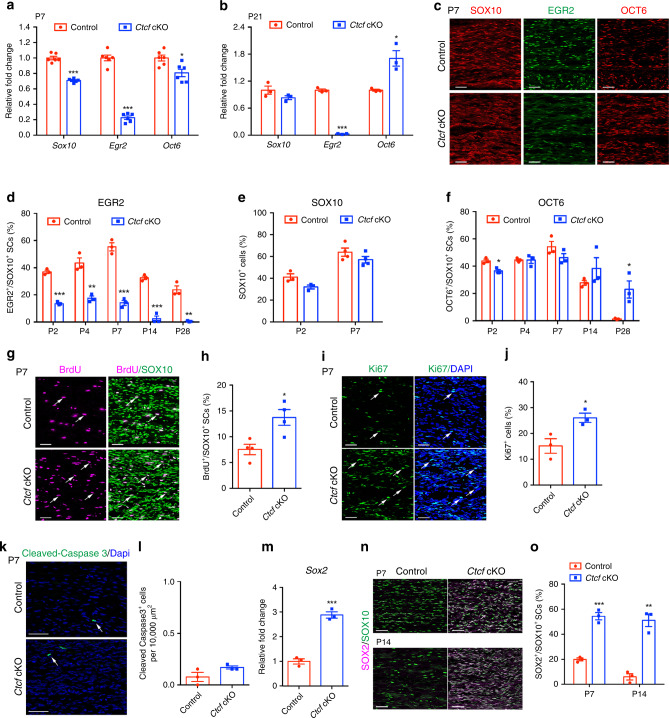
CTCF deletion in SCs inhibits SC differentiation and myelination. **a**, **b** qRT-PCR analysis of promyelinating transcriptional regulators in control and *Ctcf* cKO mice sciatic nerves at **a** P7 and **b** P21. *n* = 6 animals/genotype for P7 and *n* = 3 animals/genotype for P21, **a** *P*_(*Sox10*)_ = 3.03E-07, *P*_(*Egr2*)_ = 6.92E-09, *P*_(*Oct6*)_ = 0.0105; **b** *P*_(*Sox10*)_ = 0.171, *P*_(*Egr2*)_ = 4.97E-06, *P*_(*Oct6*)_ = 0.0152. **c** Immunolabeling of SOX10, EGR2, and OCT6 in P7 control and *Ctcf* cKO sciatic nerves (*n* = 3 animals/genotype). Scale bars: 50 μm. **d–f** Quantification of **d** EGR2^+^/SOX10^+^ cells, **e** SOX10^+^ cells, and **f** OCT6^+^/SOX10^+^ cells at different stages. *n* = 4 animals/genotype for SOX10 at P7, *n* = 3 animals/genotype for others, **d** *P*_(P2)_ = 7.27E-05, *P*_(P4)_ = 0.00331, *P*_(P7)_ = 0.000294, *P*_(P14)_ = 0.00023, *P*_(P28)_ = 0.00126; **e** *P*_(P2)_ = 0.056, *P*_(P7)_ = 0.20; **f** *P*_(P2)_ = 0.011, *P*_(P4)_ = 0.99, *P*_(P7)_ = 0.18, *P*_(P14)_ = 0.27, *P*_(P28)_ = 0.026. **g** Immunolabeling and **h** analysis of BrdU and SOX10 in P7 control and *Ctcf* cKO sciatic nerves. Arrows indicate SOX10^+^/BrdU^+^ SCs. Scale bars: 50 μm. *n* = 4 animals/genotype, *P* = 0.014. **i** Immunolabeling and **j** quantification of Ki67 in P7 control and *Ctcf* cKO sciatic nerves. Arrows indicate Ki67^+^ cells. Scale bars: 50 μm. *n* = 3 animals/genotype, *P* = 0.03. **k** Immunolabeling and **l** quantification of cleaved-caspase 3 in P7 control and *Ctcf* cKO sciatic nerves. Arrows indicate Cleaved-Caspase 3^+^ SCs. Scale bars: 100 μm. *n* = 3 animals/genotype, *P* = 0.12. **m** qPCR analysis of *Sox2* in P7 control and *Ctcf* cKO sciatic nerves. *n* = 3 animals/genotype, *P* = 0.00032. **n**, **o** Immunolabeling (**n**) and quantification of SOX2 and SOX10 (**o**) in P7 and P14 control and *Ctcf* cKO sciatic nerves. Scale bars: 50 μm. *n* = 3 animals/genotype, *P*_(P7)_ = 0.0004, *P*_(P14)_ = 0.0013. Data are presented as means ± SEM., *P < 0.05, **P < 0.01, ***P < 0.001, two-tailed unpaired Student’s *t*-test. Source data are provided as a Source Data file.

The SC myelination process is regulated by coordinated actions of positive and negative transcription factors^11,17^. In *Ctcf* cKO sciatic nerves, we detected markedly reduced levels of myelination-promoting gene *Egr2*, at P7 and P21 (Fig. 4a, b). At P7 *Sox10* and *Oct6* levels were reduced compared with controls (Fig. 4a). At P21, *Sox10* expression was comparable to the control, but *Oct6* expression was increased (Fig. 4b). The drastic reduction of *Egr2* concomitant with upregulation of the early differentiating factor *Oct6* suggests that the SC differentiation process is stagnated at the promyelinating stage in the absence of CTCF.

Consistent with transcript levels, EGR2 expression in *Ctcf* cKO mice was abolished throughout postnatal 4 weeks (Fig. 4c, d). The number of SOX10^+^ SCs in mutant nerves was comparable to that in control nerves at P2 and slightly lower at P7 (Fig. 4c, e), which is likely due to the overall reduction in differentiated SCs. However, the proportion of OCT6^+^ SCs was higher in *Ctcf* cKO nerves at P14 and P28 (Fig. 4c, f), suggesting that they were stalled at the promyelinating stage in *Ctcf* cKO nerves. This is consistent with a higher SC proliferation rate in *Ctcf* cKO nerves revealed by Ki67 and 5-bromo-2’-deoxyuridine (BrdU) incorporation (Fig. 4g–j).

We did not detect significant alteration in SC apoptosis in *Ctcf* mutants as determined by cleaved-caspase 3 in sciatic nerves at P7 (Fig. 4k, l). In addition, SOX2 expression was upregulated in sciatic nerves of *Ctcf* cKO mice at P7 and P14 (Fig. 4m–o). This is consistent with the role of SOX2 as a negative regulator of myelination^9^. Our findings indicate that *Ctcf* deletion arrests SCs at their promyelinating stage by blocking their transition into EGR2^+^ differentiated SCs.

### CTCF ablation in SCs blocks myelin regeneration after injury

**Fig. 5:**
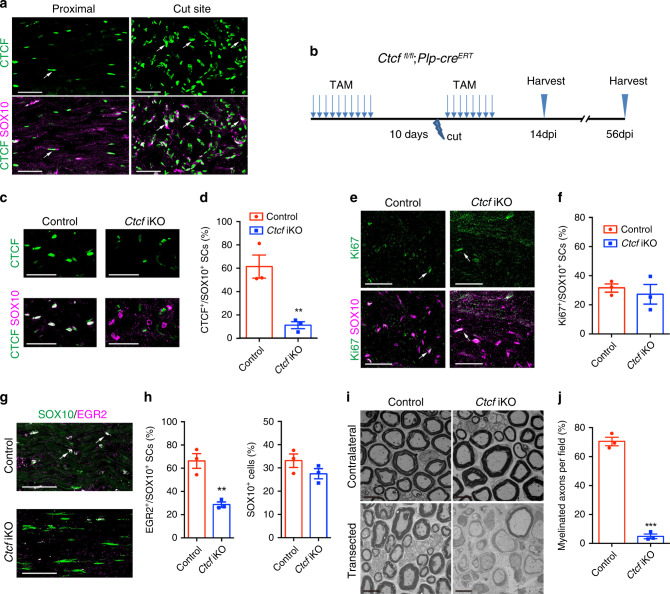
CTCF is required for SC differentiation during nerve repair. **a** Immunolabeling for CTCF and SOX10 in uninjured (proximal) and regenerating region of sciatic nerves of control mice at 14 dpi (*n* = 3 animals/genotype). Arrows indicate SOX10^+^/CTCF^+^ SCs. Scale bars: 50 μm. **b** A diagram showing the nerve transection scheme. Mice were treated with TAM via i.p. for 10 days, after 10 days, nerves were cut, and mice were then given TAM for 8 days, and nerves were analyzed at dpi 14 and 56. **c** Immunolabeling for CTCF and SOX10 in regenerating regions of control and *Ctcf* iKO sciatic nerves 14 dpi (*n* = 3 animals/genotype). Scale bars: 50 μm. **d** Proportion of CTCF^+^ SCs in the regenerating regions of 14 dpi control and *Ctcf* iKO sciatic nerves (*n* = 3 animals/genotype). *P* = 0.0083. **e** Immunolabeling for Ki67 and SOX10 in the regenerating regions of 28 dpi control and *Ctcf* iKO sciatic nerves (*n* = 2 animals/genotype). Arrows indicate representative SOX10^+^/Ki67^+^ SCs. Scale bars: 50 μm. **f** Proportion of Ki67^+^ SCs in the regenerating regions of 14 dpi control and *Ctcf* iKO sciatic nerves (*n* = 3 animals/genotype). *P* = 0.58. **g** Immunolabeling of SOX10 and EGR2 in the regenerating regions of 14 dpi control and *Ctcf* iKO sciatic nerves (*n* = 3 animals/genotype). Arrows indicate representative SOX10^+^/EGR2^+^ SCs. Scale bars: 50 μm. **h** Proportion of (left) EGR2^+^ over SOX10^+^ cells and (right) SOX10^+^ cells in the regenerating regions of 14 dpi control and *Ctcf* iKO sciatic nerves (*n* = 3 animals/genotype). *P*_(left)_ = 0.005, *P*_(right)_ = 0.19. **i** EM images of transverse sections of control and *Ctcf* iKO 8 weeks after transection (*n* = 3 animals/genotype). Scale bar: 6 μm. **j** Proportion of myelinated axons from EM images of control vs. *Ctcf* iKO 8 weeks after injury (*n* = 3 animals/genotype). *P* = 4.26E-05. Data are presented as means ± SEM., **P < 0.01, ***P < 0.001, two-tailed unpaired Student’s *t*-test. Source data are provided as a Source Data file.

Although CTCF was maintained at minimal levels in adult sciatic nerves, it was highly upregulated in regenerating SCs after injury (Fig. 5a). Inactivation of *Ctcf* in SCs of adult mice using *Plp-creERT2* did not alter sciatic nerve morphology or myelin sheath thickness, however (Supplementary Fig. 1). To determine whether CTCF is required for SC remyelination, we performed peripheral nerve transection in adult control and *Ctcf* iKO mice with or without tamoxifen treatment (Fig. 5b). After transection, SCs undergo dedifferentiation and proliferate, reattach the distal stumps, and establish a tissue bridge that guides regenerating axons to the distal stump, followed by SC redifferentiation and remyelination^17^. Tamoxifen administration prior and postinjury ablated CTCF expression in nearly all SCs from transected nerves in the *Ctcf* iKO mice (Fig. 5c, d).

For histological analyses, we harvested sciatic nerves 14 days (dpi 14) after axotomy to assess SC differentiation. In the regenerating region of sciatic nerves, the percentage of proliferative SCs (Ki67^+^SOX10^+^) was indistinguishable between *Ctcf* iKO and control mice (*Ctcf*^*loxP/loxP*^ or *Ctcf*^*loxP/+*^*;Plp1-creERT*) (Fig. 5e, f). In contrast, SC redifferentiation was severely impaired in *Ctcf* iKO as indicated by a substantial decrease in EGR2^+^ differentiated SCs (Fig. 5g, h). EM analysis shows that a majority of axons were remyelinated at the regenerating site in control nerves 8 weeks postinjury, whereas *Ctcf* iKO mice exhibited significantly fewer remyelinated axons and thinner myelin sheath thickness (Fig. 5i, j). EGR2^+^ differentiated SCs were substantially diminished in *Ctcf* iKO mice despite the presence of SC-lineage cells (Fig. 5h), suggesting that *Ctcf*-mutant SCs were obstructed at the promyelinating stage. Taken together, our data show that CTCF is required for SC remyelination during peripheral nerve regeneration.

### CTCF regulates the transcriptional program in SCs

**Fig. 6:**
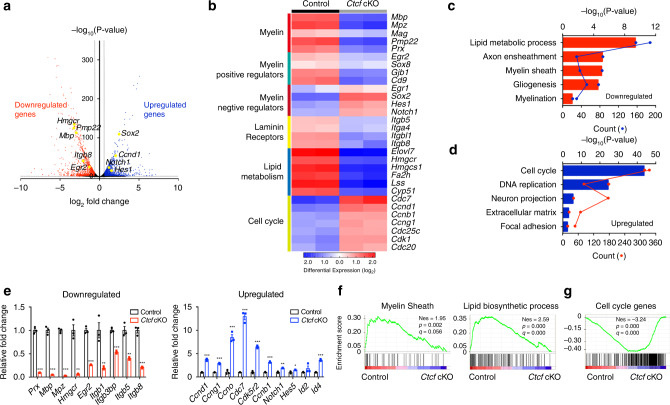
CTCF regulates the transcriptional program of SC differentiation. **a** Volcano plot of transcriptome profiles of control and *Ctcf* cKO sciatic nerves (*n* = 2 animals/genotype). Red and blue dots represent significantly downregulated and upregulated genes in *Ctcf* cKO nerves compared to the control, respectively (*P* < 0.05, fold-change > 1.5). **b** Heatmap of representative genes and their categories differentially expressed in control and *Ctcf* cKO sciatic nerves (*n* = 2 animals/genotype). **c**, **d** Bar plots of gene ontology analysis of genes **c** downregulated and **d** upregulated genes in *Ctcf* cKO sciatic nerves compared with control nerves. Each dot (connected by lines) represents the gene count of the corresponding biological function categories. *n* = 2 independent tissues/genotype. **e** qPCR analysis of genes related to SC development that are decreased (left) and increased (right) in *Ctcf* cKO sciatic nerves relative to control. **f** GSEA enrichment scores for myelin sheath (left) and lipid biosynthetic process (right) gene sets in control and *Ctcf* cKO sciatic nerves. **g** GSEA enrichment scores for cell-cycle gene sets in control and *Ctcf* cKO sciatic nerves. Data are presented as means ± SEM., ***P < 0.001, **P < 0.01, *P < 0.05, *n* = 3 animals/genotype; two-tailed unpaired Student’s *t*-test, *P*_*(Prx)*_ = 2.6e-05, *P*_*(Mbp)*_ = 4.9E-05, *P*_*(Mpz)*_ = 5.3E-06, *P*_*(Hmgcr)*_ = 0.0014, *P*_*(Egr2)*_ = 8.6E-05, *P*_*(Itgb1)*_ = 0.008, *P*_*(Itgb3bp)*_ = 0.00022, *P*_*(Itgb5)*_ = 0.0021, *P*_*(Itgb8)*_ = 0.00017, *P*_*(Ccnd1)*_ = 7.4E-05, *P*_*(Ccng1)*_ = 5.1E-05, *P*_*(Ccno)*_ = 0.0004, *P*_*(Cdc7)*_ = 6.4E-05, *P*_*(Cdk5r2)*_ = 1.6E-05, *P*_*(Ccnb1)*_ = 3.2E-05, *P*_*(Notch1)*_ = 0.00102, *P*_*(Hes5)*_ = 0.028, *P*_*(Id2)*_ = 0.23, *P*_*(Id4)*_ = 3.3E-05. Source data are provided as a Source Data file.

To investigate the potential mechanisms of CTCF regulation of SC myelination, we performed RNA-seq of control and *Ctcf* cKO sciatic nerves at P7. Deletion of *Ctcf* in sciatic nerves significantly upregulated or downregulated a number of genes (>1.5-fold change, *P*-value < 0.05; Fig. 6a). Genes associated with SC myelination, laminin receptors, and lipid metabolism pathways such as *Mpz*, *Pmp22*, *Egr2*, *Itgb8*, *Hmgcr*, and *Lss* were downregulated in *Ctcf* cKO sciatic nerves, consistent with the dysmyelination phenotype of *Ctcf* cKO mice. Genes that were upregulated are associated with negative regulators of myelination and cell-cycle progression including *Sox2*, *Notch1*, and *Ccnd1* (Fig. 6b). Gene ontology analysis revealed that the functions of the genes most significantly downregulated were particularly enriched for lipid metabolic process, myelin sheath, and gliogenesis (Fig. 6c), whereas those most significantly upregulated genes were categorized into cell-cycle control, DNA replication, and extracellular matrix (Fig. 6d). qRT-PCR showed that the genes involved in lipid metabolic processes, myelin sheath formation, and SC differentiation were expressed at lower levels in the *Ctcf* cKO sciatic nerve, whereas differentiation inhibitors, such as *Notch1*, *Id2*, and *Hes5*, were expressed at higher levels (Fig. 6e). Gene Set Enrichment Analysis (GSEA) also confirmed that genes categorized as involved in myelinogenesis were downregulated and that cell-cycle genes were upregulated (Fig. 6f, g).

**Fig. 7:**
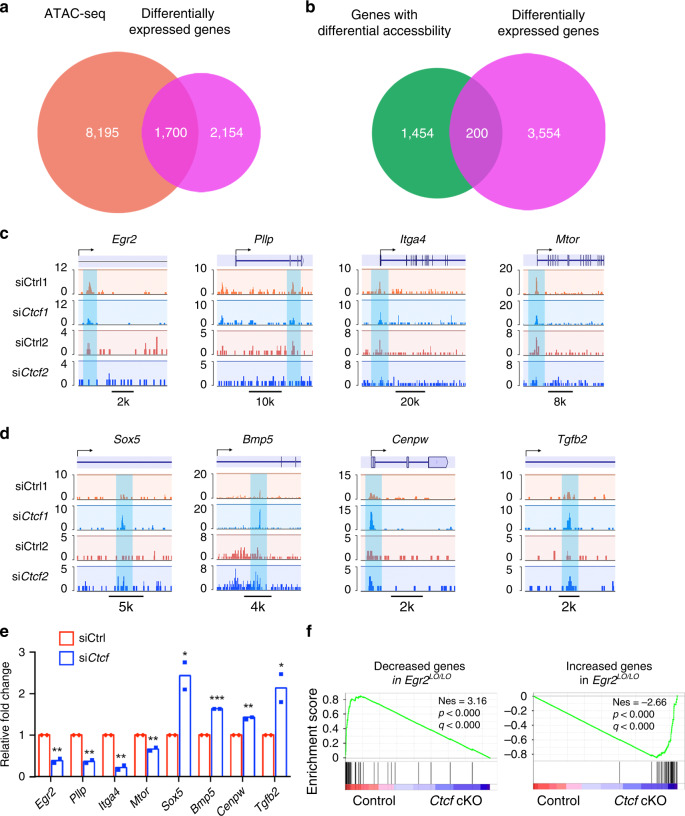
CTCF regulates chromatin accessibility during SC differentiation. **a**, **b** Venn diagram showing the overlap between the genes located in open chromatin sites in rat SCs (**a**) or the genes with differential chromatin accessibility (**b**) by ATAC-seq with genes differentially expressed genes between control and *Ctcf* cKO sciatic nerves. **c**, **d** Representative ATAC-seq signals around the *Egr2* MSE and myelination-related gene loci (**c**), as well as SC negative- or proliferation-related genes (**d**) in control or *siCtcf*-treated rat SCs, *n* = 2 biological replicates for control and si*Ctcf* SCs. **e** Relative fold-change of ATAC-seq peaks in panel **c** and **d**. *n* = 2 biological replicates for control and si*Ctcf* SCs. **f** GSEA enrichment scores for genes downregulated (left) or upregulated (right) genes in *Egr2* ^Lo/Lo^ nerves from published data^34^. NES, normalized enrichment score. Data are presented as means ± SEM., *P < 0.05, **P < 0.01, ***P < 0.001; one-tailed unpaired Student’s *t*-test, *P*_*(Egr2)*_ = 0.0033, *P*_*(Pllp)*_ = 0.0014, *P*_*(Itga4)*_ = 0.0021, *P*_*(Mtor)*_ = 0.0053, *P*_*(Sox5)*_ = 0.024, *P*_*(Bmp)*_ = 4E-07, *P*_*(Cenpw)*_ = 0.0017, *P*_*(Tgfb2)*_ = 0.039. Source data are provided as a Source Data file.

To determine the impact of *Ctcf* depletion on chromatin accessibility, we performed an assay for transposase-accessible chromatin (ATAC-seq) assay^31^ in rat SCs grown under differentiation conditions treated with control siRNA or with si*Ctcf*. We intersected the genes located in open chromatin in rat SCs treated with the control siRNA with those genes differentially expressed in *Ctcf* cKO sciatic nerves and identified ~1700 genes (Fig. 7a). Among them, ~200 genes were accompanied with differential accessibility upon *Ctcf* siRNA (Fig. 7b and Supplementary Data 1). Compared with control SCs, chromatin accessibility in the promoters or enhancers of myelination-associated genes *Egr2*, *Pllp*, *Itga4*, and *Mtor* decreased in *siCtcf*-treated SCs (Fig. 7c, e) whereas it increased in the promoters of SC negative or proliferation-regulatory genes, such as *Sox5*, *Bmp5*, *Cenpw*, and *Tgfb2*^32,33^ (Fig. 7d, e). In addition, GSEA analysis of the altered genes in *Egr2-*deficient mice^34^ compared with *Ctcf* cKO mice shows that the gene expression of *Egr2* hypomorphic (*Egr2*^Lo/Lo^) mice^34^ were corelated with those in *Ctcf* cKO mice (Fig. 7f), suggesting that the phenotype of CTCF knockout is likely driven by the loss of EGR2 functions.

We next examined expression of the transcriptional factors such as NFATc3/4, YAP1, and TEADs that are known to regulate *Egr2*^35,36^. Expression of *Nfatc3*, *Nfatc4*, *Yap1*, and *Tead1* was not altered substantially in *Ctcf-*deficient nerves, while expression of individual Tead2-4 mRNAs exhibited a differential response to *Ctcf-*deficiency, suggesting that CTCF may have a distinct function in the regulation of TEAD/YAP family members (Supplementary Fig. 2). Together, these results suggest that CTCF modulates chromatin accessibility of genes associated with the SC differentiation and myelination program.

### CTCF interacts with PRC2 to repress SC differentiation

**Fig. 8:**
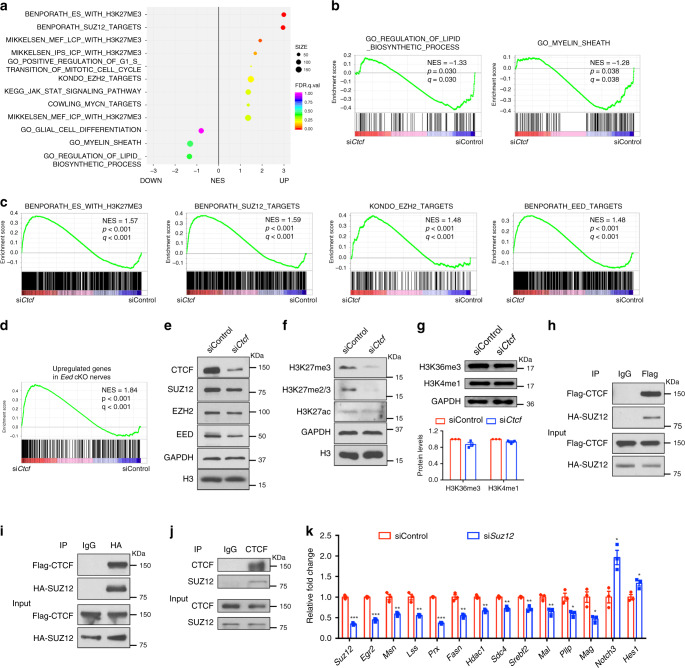
CTCF cooperates with PRC2 complex to regulate SC differentiation. **a** GSEA enrichment scores for sets of genes differentially regulated in rat SCs treated with si*Ctcf* or control siRNA. *n* = 3 independent experiments. **b** GSEA enrichment scores for genes involved in lipid biosynthetic process (left) and myelin sheath (right) in SCs treated with control or si*Ctcf*. **c** GSEA enrichment scores for genes modified with H3K27me3 and for genes targeted by SUZ12, EZH2, or EED in SCs treated with control or si*Ctcf*. **d** GSEA enrichment scores for genes upregulated genes in *Eed* cKO nerves from published data^37^. NES, normalized enrichment score. **e–g** Immunoblotting for **e** PRC2 complex and CTCF proteins and for **f**, **g** histones with indicated modifications in SCs treated with control or si*Ctcf*. **e**, **f** *n* = 2 independent experiments; **g** *n* = 3 independent experiments. **h–j** Co-immunoprecipitation of **h**, **i** HA-SUZ12 with Flag-CTCF from extracts of transiently transfected HEK293T cells or of **j** endogenous SUZ12 with CTCF from rat SCs. *n* = 2 independent experiments. **k** qRT-PCR analysis showing *Suz12* and differentiation-related and myelin-related gene expression in rat SCs transfected with control siRNA or with *Suz12*-targeted siRNA (*n* = 3 independent experiments) and induced to differentiate for 9 h. Data are presented as means ± SEM., ***P < 0.001, **P < 0.01, *P < 0.05; two-tailed unpaired Student’s *t*-test, *P*_(*Suz12*)_ = 3.8E-05, *P*_(*Egr2*)_ = 3.3E-05, *P*_(*Msn*)_ = 0.0045, *P*_(*Lss*)_ = 0.0031, *P*_(*Prx*)_ = 1.5E-06, *P*_(*Fasn*)_ = 0.0019, *P*_(*Hdac1*)_ = 0.0019, *P*_(*Sdc4*)_ = 0.0015, *P*_(*Srebf2*)_ = 0.0026, *P*_(*Mal*)_ = 0.008, *P*_(*Pllp*)_ = 0.018, *P*_(*Mag*)_ = 0.017, *P*_(*Notch3*)_ = 0.012, *P*_(*Hes1*)_ = 0.02. Source data are provided as a Source Data file.

GSEA revealed that genes related to myelin and lipid biosynthesis were downregulated, while genes repressed by H3K27me3 and PRC2 were upregulated in CTCF-deficient SCs (Fig. 8a–c). Consistent with the previous study^37^, PRC2/EED targets, which are upregulated in *Eed* cKO nerves, were enriched in si*Ctcf* SCs (Fig. 8d). This suggests that CTCF depletion leads to a downregulation of PRC2 activity, which acts as a transcriptional repressor by catalyzing methylation of histone H3 on Lys27 (H3K27me3) on gene regulatory elements^38,39^.

Although the levels of PRC2 complex components SUZ12, EZH2, and EED were only modestly downregulated in si*Ctcf*-treated SCs (Fig. 8e), the levels of H3K27me3 and H3K27me2/3 were substantially reduced in si*Ctcf*-treated cells compared to the control (Fig. 8f), whereas other epigenetic modification marks, such as H3K36me3, H3K4me1, and H3K27ac, were comparable between control and si*Ctcf*-treated SCs (Fig. 8f, g). This suggests that downregulation of CTCF selectively diminishes PRC2 complex activity in SCs.

SUZ12 acts as scaffold for PRC2 complex integrity and functions^39–41^. PRC2 components may provide distinct molecular cues in different contexts^38^. To test whether CTCF and SUZ12 form a complex, we transiently transfected HEK293T cells with vectors expressing flag-tagged CTCF and HA-tagged SUZ12; in a co-immunoprecipitation assay we detected CTCF in a complex with SUZ12 (Fig. 8h, i). Furthermore, endogenous SUZ12 was co-immunoprecipitated with CTCF in differentiated SCs (Fig. 8j), consistent with previous study in other cell types^41^. These results raise the possibility that CTCF coordinates with PRC2 to mediate gene silencing during SC differentiation.

To evaluate the functional role of the SUZ12-PRC2 complex, we knocked down *Suz12* expression by siRNA in SCs. Similar to the CTCF-deficient SCs, *Suz12* silencing downregulated SC differentiation-associated genes such as *Egr2, Prx, Fasn, Srebf2, Mal*, *Pllp*, and *Mag*, while upregulating differentiation-inhibitory genes, such as *Notch3* and *Hes1* (Fig. 8k). This suggests that SUZ12-mediated PRC2 activity is required for proper SC differentiation.

### CTCF recruits SUZ12 to regulate SC differentiation

**Fig. 9:**
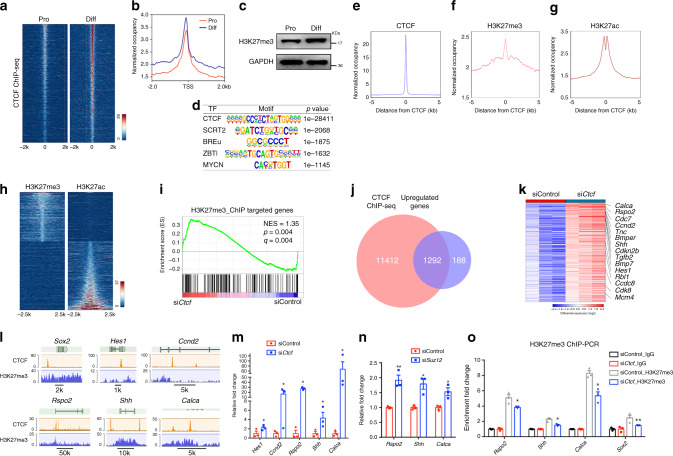
CTCF and SUZ12 regulate transcriptomic dynamics during SC differentiation. **a** Heatmap of CTCF ChIP-seq peaks from proliferating and differentiated SCs. *n* = 1 in each condition. **b** ChIP-seq enrichment around TSS regions in proliferative and differentiated SCs. **c** Immunoblotting for H3K27me3 in proliferating and differentiating SCs. *n* = 2 independent experiments. **d** Enriched motifs of transcription factors (TF) in the CTCF-bound regions. **e–g** ChIP-seq enrichment around CTCF binding regions for **e** CTCF, and **g** H3K27ac in rat SCs, **f** H3K27me3 in rat sciatic nerves. **h** Heatmap for H3K27me3 and H3K27ac. The sites are ranked in ascending order of H3K27ac intensity. *n* = 1 in each condition. **i** GSEA enrichment for genes with H3K27me3 peaks associated with TSSs in siControl or si*Ctcf* rat SCs. NES, normalized enrichment score. **j** Overlap between CTCF-bound and upregulated genes differentially expressed in rat SCs treated with control siRNA or si*Ctcf*. **k** Heatmap of CTCF-targeted upregulated genes in rat SCs treated with control siRNA or si*Ctcf*. *n* = 3 in each condition. **l** Tracks for the indicated genes with ChIP-seq of CTCF from rat SCs and of H3K27me3 from rat sciatic nerves. *n* = 1 in each condition with 20 million cells. **m** Expression of the indicated genes in RNA-seq dataset from rat SCs treated with control siRNA or si*Ctcf*. *n* = 3 independent experiments, *P*_(*Hes1*)_ = 0.011, *P*_(*Ccnd2*)_ = 0.01007, *P*_(*Rspo2*)_ = 0.0105, *P*_(*Shh*)_ = 0.019, *P*_(*Calca*)_ = 0.041. **n** qRT-PCR for the indicated genes in rat SCs treated with control siRNA or si*Suz12*. *n* = 3 independent experiments, *P*_(*Rspo2*)_ = 0.006, *P*_(*Shh*)_ = 0.012, *P*_(*Calca*)_ = 0.02. **o** ChIP-qPCR for H3K27me3 at the promoters of the indicated genes in rat SCs treated with control siRNA or si*Ctcf*. IgG were normalized to 1; *n* = 3 independent experiments, *P*_(*Rspo2*)_ = 0.039, *P*_(*Shh*)_ = 0.011, *P*_(*Calca*)_ = 0.039, *P*_(*Sox2*)_ = 0.0094. Data are presented as means ± SEM, *P < 0.05, **P < 0.01, ***P < 0.001, two-tailed unpaired Student’s *t*-test. Source data are provided as a Source Data file.

To identify genes directly targeted by CTCF, we performed chromatin immunoprecipitation sequencing (ChIP-Seq) in proliferating and differentiating SCs. The signals of CTCF peaks within ±2 kb elements proximal to transcriptional start sites appeared to have an increase in differentiating SCs compared to proliferative SCs (Fig. 9a, b), accompanied by increased levels of H3K27me3 in differentiating SCs (Fig. 9c). To identify consensus sequence motifs associated with CTCF targeted sites using the HOMER program, we observed the binding motifs for CTCF, SCRT2, BREU, ZBTB3, and MYCN (Fig. 9d). In ~5.8% of CTCF-occupied regions, H3K27me3 peaks were observed (Fig. 9e, f, and Supplementary Data 2). In addition, ~16.8% of CTCF peaks overlapped with those of the activating histone mark H3K27ac^42^ in SCs (Fig. 9g; Supplementary Data 2). These data indicate that CTCF binding sites can be associated with either repressive or activating regulatory elements. H3K27me3 was essentially excluded from H3K27ac-enriched enhancers and promoters in SCs (Fig. 9h), it suggests that CTCF may control the expression of distinct differentiation factors to regulate SC differentiation programs.

The H3K27me3-targeted genes from ChIP-seq assays were significantly more enriched in si*Ctcf* SCs than SCs treated with control siRNAs (Fig. 9i), suggesting that *Ctcf* loss leads to a derepression of H3K27me3-targeted genes. Intersection of the CTCF-occupied genes with those differentially expressed in SCs treated with control vs. *Ctcf*-targeted siRNA revealed approximately 1292 upregulated genes that are likely to be CTCF-regulated targets (Fig. 9j). The most significantly upregulated genes from GO analysis are associated with immature SCs and include *Hes1*, encoding an effector of Notch signaling that inhibits SC myelination^16,43^, *Rspo2*, which encodes a protein that confers stemness-associated traits^44,45^, *Shh*, reflecting a reversion from mature to proliferating immature SCs^46,47^, and *Calca*, encoding calcitonin-related polypeptide that is critical for SC proliferation^48,49^ (Fig. 9k, l, and Supplementary Data 3). Transcriptomic and qRT-PCR analyses validated that expression of these genes was significantly upregulated in both *Ctcf*- and *Suz12*-silenced SCs (Fig. 9m, n).

To further determine whether H3K27me3 deposition is altered over promoters after CTCF-depletion, we performed ChIP followed by qPCR (ChIP-qPCR) for H3K27me3 in control and si*Ctcf*-treated SCs. H3K27me3 occupancy was markedly reduced at the promoters of *Rspo2, Shh, Calca*, and *Sox2* in the absence of CTCF (Fig. 9o), suggesting that CTCF cooperates with PRC2 to repress the expression of genes that prevent SC differentiation.

### CTCF-mediated chromatin looping required for EGR2 expression

**Fig. 10:**
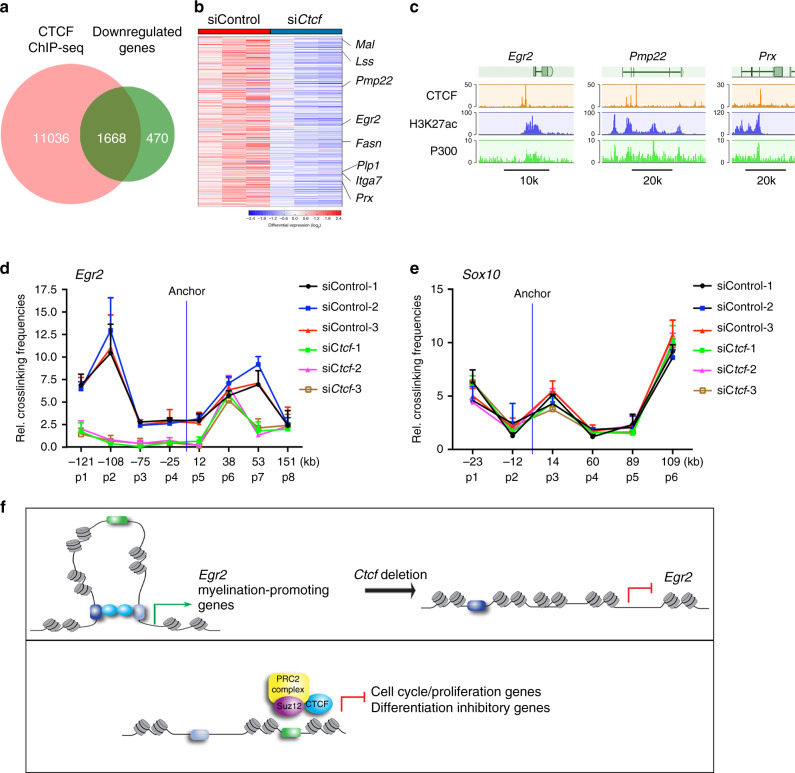
CTCF-mediated chromatin regulatory looping is necessary for EGR2 expression. **a** Venn diagrams depicting overlap between CTCF-bound genes and downregulated genes differentially expressed in rat SCs treated with control siRNA or si*Ctcf*. **b** Heatmap of CTCF-targeted genes differentially downregulated in SCs treated with si*Ctcf* compared to controls. *n* = 3 in each condition. **c** Genome browser tracks over the loci of selected myelin-related genes with ChIP-seq density mapping of CTCF, H3K27ac, and P300 from rat SCs. *n* = 1 in each condition with 20 million cells. **d** Quantitation of relative interaction frequencies between the indicated anchor site and neighboring *Egr2* genomic restriction fragments. *n* = 3 independent 3C experiments. **e** Quantitation of relative interaction frequencies between the indicated anchor site and neighboring *Sox10* genomic restriction fragments. *n* = 3 independent 3C experiments. **f** A model depicting a dual mode of action by CTCF in promotion of SC differentiation: CTCF stabilizes chromatin loops involving promotor-enhancer interactions to activate expression of promyelinating genes such as *Egr2* (left panel) and forms a transcriptional co-repressor complex with SUZ12-PRC2 (right panel) to inhibit expression of genes such as *Sox2* that encode factors that inhibit differentiation and cell-cycle/proliferation regulators. Data are presented as means ± SEM. Source data are provided as a Source Data file.

To identify candidate CTCF-regulated targets, we intersected the CTCF-occupied genes with those genes differentially downregulated expressed in si*Ctcf* SCs compared to controls (Fig. 10a). The downregulated genes that are occupied by CTCF were associated with SC differentiation and lipid biogenesis including *Egr2*, *Pmp22*, and *Lss* (Fig. 10b, c).

The loss of CTCF caused a drastic reduction in expression of the critical SC myelination gene *Egr2*. Since CTCF is a crucial mediator of enhancer-promoter interactions and long-range genomic landscaping^50,51^, we tested whether CTCF regulates promoter-enhancer looping on the *Egr2* locus by performing quantitative chromosome conformation capture (3C) in control and si*Ctcf*-treated SCs across ~270 kilobases of the *Egr2* genomic locus on rat chromosome 20^51^. 3C assays allow capture of long-range interactions between *cis*-acting elements^51,52^. We assessed interactions between the anchor site within the promoter region with upstream (primers p1-p4) and downstream (primers p5–p8) regulatory or enhancer regions marked by the activating histone mark H3K27ac of the *Egr2* locus in a 3C assay in SCs (Supplementary Fig. 3a). We found that the *Egr2* promoter interacted at a high frequency with the upstream p2 and downstream at p7 regulatory elements, while minimal interactions between the anchor site and other *Egr2* elements were observed (Fig. 10d). In the CTCF-deficient SCs, interactions between *Egr2* promoter and its enhancers at p1, p2, and p7 sites, were markedly reduced compared to the interaction in control cells (Fig. 10d), suggesting that CTCF is required for the enhancer-promotor interactions with the *Egr2* locus. The interaction between *Egr2* promoter and the myelin-specific element (MSE)^53^ at the p6 site (around +38 kb) does not appear to be CTCF regulated, however, the chromatin accessibility in the *Egr2* MSE is reduced in *Ctcf*-knockdown SCs (Fig. 7c, e). In contrast, CTCF downregulation does not impair the chromatin looping around the *Sox10* locus (Fig. 10e). Given the downregulation of EGR2, but not SOX10, in CTCF-deficient SCs, this suggests that the CTCF-mediated enhancer-promotor looping is required for expression of *Egr2*, but not *Sox10*.

Although depletion of CTCF drastically decreased expression of *Egr2*, the genes neighboring *Egr2* such as *Nrbf2*, and *Jmjd1c*, which are located outside of the genomic topologically associating domain during mouse neural development^54^, were not substantially affected by CTCF depletion (Supplementary Fig. 3b-d), suggesting that the role of CTCF-mediated chromatin organization is specific to the *Egr2* gene during SC differentiation.

## Discussion

Although CTCF is a regulator of chromatin organization and enhancer function^3,18,19^, little had been known regarding its function in SC development and myelin regeneration. In this study, we found that CTCF is upregulated during SC differentiation and restricts chromatin accessibility in the gene loci associated with immature SC programs while silencing differentiation-inhibitory cues through recruiting PRC2/SUZ12 repressive complexes for promyelination gene expression. *Ctcf* deletion in SCs resulted in severe hypomyelination with a complete absence of myelinating axons in peripheral nerves. The loss of *Ctcf* blocked SC differentiation and myelination at the promyelinating state but did not appear to affect immature SC survival or myelin maintenance, indicating a stage-specific function of CTCF during SC-lineage progression. Recently, a short isoform of CTCF (CTCF-s) was identified to have a noncanonical role to antagonize full-length CTCF^55^. It remains to be defined for the function of CTCF-s in SC development.

In *Ctcf*-mutant sciatic nerves, we observed drastic downregulation of the critical SC differentiation regulators such as EGR2; however, expression of early stage SC regulators such as SOX2 and OCT6 was maintained in neonatal stages and even increased at later postnatal stages. Furthermore, CTCF deficiency led to an upregulation of differentiation-inhibitory cues, such as SOX2, NOTCH, SHH, and WNT pathways. These results suggested that CTCF functions as a dual-level switch to control of SC maturation through inactivation of differentiation-inhibitory signaling while promoting activation of promyelinating factors such as EGR2 during SC development.

The chromatin organizer CTCF can function as a repressor, activator, or insulator of gene expression depending on the genomic context^3,50^. We showed that CTCF overexpression promoted SC myelination program and repressed differentiation-inhibitory pathways, whereas the depletion of CTCF led to an increase in chromatin accessibility and derepression of genes associated with SC differentiation inhibition. This indicates that CTCF limits the expression of genes specifically expressed in immature or proliferating SCs. Thus, CTCF is required for the precise balance of proliferation versus differentiation during SC development.

We found that CTCF is critical for establishing the chromatin conformation necessary for the promotor-enhancer interaction that activates expression of the key SC promyelination factor EGR2. The activities of H3K27ac-marked enhancers around the promoters or enhancers of *Egr2* were substantially increased during SC differentiation (Supplementary Fig. 4a, b). Our ChIP-seq analysis showed that CTCF targets the enhancers of SC myelination genes during SC differentiation. suggesting that CTCF may modulate the enhancer landscape for activating expression of myelination-promoting genes such as *Egr2* during SC differentiation. Intriguingly, silencing of *Ctcf* appeared to elevate the promoter or enhancer activity around SC differentiation-inhibitory genes (Supplementary Fig. 4c). Since CTCF binding sites are interwoven with enhancer elements throughout the genome, our data indicate that CTCF plays a dual role during SC differentiation, inhibiting enhancer activity of immature SC-associated genes while activating the enhancer activity of myelin-related genes.

Our 3C assessment indicated that the enhancer region of *Egr2* forms a complex looped architecture that likely brings regulatory components into proximity. This hub involving the promoter and enhancer elements may ensure high levels of *Egr2* transcription during SC differentiation. Depletion of CTCF drastically decreased expression from *Egr2* but not neighboring genes outsides of the topologically associating domain (Supplementary Fig. 3), suggesting that CTCF specifically acts to upregulate *Egr2* during SC differentiation through establishing enhancer-promoter interaction associated with its expression. These findings are consistent with the notion that TADs are evolutionarily and developmentally stable regions and preferentially interact each other within TAD^56^. It is remained to be determined whether enhancer-promoter interactions of the *Egr2* neighboring genes such as *Nrbf2* and *Jmjd1c* outside of the TAD domain of *Egr2* are altered in the absence of CTCF. Whether and how CTCF regulates organizational architectures that shape the three-dimensional regulatory elements of other SC differentiation-promoting or inhibitory genes remain to be defined. Nonetheless, our results suggest a mechanism by which CTCF-mediated long-range interactions achieve cell-type and target-specific gene expression (Fig. 10f).

The PRC2 repressive complex is the sole histone methyltransferase that catalyzes the H3K27me3 mark^41,57^. Our data suggest that CTCF binding is critical for SUZ12-PRC2 recruitment onto the regulatory elements of genes that inhibit SC differentiation. PRC2 functions mainly to maintain rather than establish patterns of gene repression^39^, thus we reason that PRC2 functions to safeguard the SC differentiation program. In support of this hypothesis, the PRC2 component EED has been shown to be crucial for SC proliferation and differentiation and prevent alternative lineages in response to nerve injury by silencing differentiation-inhibitory and cell-cycle regulators^37,58^. EED does not, however, influence early development of myelin^37,58^. SUZ12 acts as structural scaffold for core PRC2 complex integrity and chromatin binding, but also its interactions with accessory cofactors that define distinct functions of PRC2 subcomplexes^39,40^. The distinct functions of individual PRC2 subunits have been reported in other contexts^39,59^. We showed that depletion of SUZ12 reduced expression of genes that induce SC differentiation, suggesting that SUZ12 might have activities distinct from those of EED in regulation of the SC myelination program. Of note, nonsense and inactivating mutations in PRC2 subunits like EED or SUZ12 are observed in malignant peripheral nerve sheath tumors^60^, suggesting that the loss of PRC2 function contributes to the proliferation of immature SCs during malignancy.

Genome-wide ChIP-seq revealed that H3K27me3 binding sites and CTCF binding sites overlap in genes encoding a subset of factors that inhibit differentiation, suggesting that CTCF may recruit the SUZ12/PRC2 complex to repress expression of certain genes. CTCF-mediated SUZ12/PRC2 recruitment has been shown to silence gene transcription^41,61^. Transcriptome profiling of SCs depleted of CTCF revealed a derepression of H3K27me3 target genes, suggesting that CTCF is required for the repressive activity of the PRC2 complex on these genes. The recruitment of SUZ12/PRC2 may stabilize CTCF-dependent genomic loops in genes marked with H3K27me3 to maintain their repressive states. Although CTCF may recruit other transcriptional repressors such as HDAC/SIN3a^62^, our data suggest that CTCF can promote SC differentiation by modulating the recruitment of the SUZ12/PRC2 repressive complex to silence the differentiation-inhibitory network (Fig. 10f). It is possible that the interaction between CTCF and PRC2 complexes such as SUZ12 could be indirect through other interacting mediators. Together, our studies demonstrate a critical function of CTCF-dependent chromatin architecture and local chromatin environments in control of gene expression programs necessary for SC myelination and remyelination. Although CTCF regulation is expected to be ubiquitous, our data demonstrate a temporal specificity of CTCF that is transiently required to establish a differentiated state during SC myelination. Our study revealed that expression of CTCF is upregulated during remyelinating phases after nerve injury, and is crucial for SC remyelination after nerve transection. The proliferation of SCs was not affected in the regenerating region of CTCF-deficient nerves, suggesting that CTCF may not be required for the generation of repair cells^47^, despite its essential role in normal remyelination after injury. This phenotype is similar to that of the transcriptional repressor ZEB2 in nerve repair^33,63^. However, CTCF does not appear to regulate ZEB2 expression (Supplementary Fig. 5), suggesting that CTCF may selectively control only certain promyelin regulators such as EGR2 to regulate myelin repair. Thus, manipulating CTCF function and activity in SCs may provide a therapeutic means of reversing adverse neuropathies.

## Methods

### Animals

*Ctcf*^*loxP/loxP*^ mice, generated from *Ctcf*^tm1a(EUCOMM)Wtsi^ mice breeding with ACT-FLPe mice (Jackson Laboratory, USA), were crossed with mice carrying Dhh-cre^10^ to obtain *Ctcf*^*loxP*/+;^Dhh-cre^+/−^ mice, which were then bred with *Ctcf*^*loxP/loxP*^ mice to generate control (*Ctcf*^*loxP/+;*^Dhh-cre^+/−^) and *Ctcf* cKO offspring (*Ctcf*^*loxP/loxP*^; Dhh-cre^+/−^). Littermates *Ctcf*^*loxP/loxP*^ or *Ctcf*^*loxP*/+;^Dhh-cre^+/−^ mice were used as controls. Recombination, perinatally or after sciatic nerve transection, was achieved by crossing *Ctcf*^*loxP/loxP*^ mice with the inducible Cre recombinase CreERT2 under the control of the Plp1 promoter (Plp1-creERT) followed by tamoxifen injection^30^. Animals of either sex were used in the study and littermates were used as controls. The mouse strains used in this study were generated and maintained on a mixed C57Bl/6;129 Sv background and housed in a vivarium with a 12-h light/dark cycle. Ambient temperature (22 °C) and 30–70% humidity was maintained. No more than four adult mice were housed in the same cage. The animal experiments were conducted with both genders. All animal use and studies complied with all relevant ethical regulations and were approved by the IACUC (Institutional Animal Care and Use Committee) at the Cincinnati Children’s Hospital Medical Center, USA.

### Isolation, growth, and differentiation of primary rat SCs

Rat SCs were isolated from sciatic nerves of newborn rats (1–2 d old). Sciatic nerves were harvest and dissociated by Trypsin-Collagenase (Gibco, 25200-056), then cells were treated with SC medium supplemented with Ara-C (Sigma, C6645) for 3 days and washed out fibroblasts. After treating with SC medium supplemented with anti-Thy-1.1 antibody (Serotec, MCA04G), cells were wash with HBSS (Gibco, 14170-112) to remove the remaining contaminating fibroblasts. Isolated SCs were grown routinely in DMEM/10% FBS (Life Technologies), supplemented with 10 ng ml^−1^ Neuregulin 1 (Nrg1; R&D Systems, 396-HB) and 5 μM forskolin (Sigma, F6886) plus L-glutamine and penicillin/streptomycin, hereafter termed SC proliferation medium. Cells between passages 2 and 6 were used in all experiments. >95% SC purity was achieved, assessed by SOX10 and S100β staining. To initiate differentiation, SCs were washed three times with DMEM and then cultured in differentiation medium containing DMEM/0.5% FBS and 1 mM dibutyryl cyclic AMP (Sigma, D0627) with L-glutamine and penicillin/streptomycin, for the length of time indicated in the text, depending on the assays used. All tissue culture containers and coverslips were coated with 50 μg ml^−1^ poly-L-lysine (Sigma, P-7890) in PBS for at least 30 min at room temperature and then rinsed in distilled water for three times. Purified rat SCs seeded on coverslips were fixed in 4% paraformaldehyde (PFA) for 15 min and washed in 1× PBS four times before immunofluorescence staining.

### Immunostaining and electron microscopy

The sciatic nerves of mice at defined ages were dissected and fixed for 30 min in 4% PFA, embedded in OCT, cryoprotected in 25% sucrose and sectioned at 9 μm as longitudinal sections by using cryostat. For BrdU pulse labeling, animals at P7 were injected subcutaneously with 100 mg BrdU kg-1 body weight 2 h before sciatic nerve collection. For immunostaining, we used antibodies to CTCF (rabbit, Cell Signaling, #3418, 1:600), SOX10 (goat, Santa Cruz Biotechnology, sc-17342, 1:500; rabbit, Abcam; ab155279, 1:500), Oct6 (goat, Santa Cruz Biotechnology, sc-11661, 1:200), EGR2 (rabbit, Santa Cruz Biotechnology, sc-20690, 1:200), MBP (goat, Santa Cruz Biotechnology, sc-13914, 1:500), SOX2 (goat, Santa Cruz Biotechnology, sc-17320, 1:200), Ki67 (rabbit, Thermo Scientific, RM-9106, 1:500), BrdU (rat, Abcam, ab6326, 1:200), cleaved-caspase 3 (rabbit, Cell Signaling, #9661, 1:200), NF-M (rabbit, Millipore, AB1987, 1:200). Secondary antibodies conjugated to Cy2, Cy3, or Cy5 were from Jackson ImmunoResearch Laboratories catalog numbers 705-165-147, 705-225-147, 711-225-152, 711-165-152, 711-175-152, 715-165-150, and 712-165-150 (1:500). All images were acquired using Nikon C2 confocal microscope. The threshold for the quantifications was based on the strong visual appearance of immunostaining signal of quantified cells compared with the background in a blinded manner.

For electron microscopy, mice were perfused with 4% PFA, 2.5% glutaraldehyde in 0.1 M sodium cacodylate buffer, pH 7.2. Sciatic nerves were dissected and fixed in the same fixative solution overnight. Nerves were rinsed in PBS, postfixed in 1% OsO4 in PBS for 1 h, dehydrated in graded ethanol, infiltrated with propylene oxide and embedded in Epon. Semi-thin sections were stained with toluidine blue, and thin sections were stained with lead citrate. The morphometric measurements of axonal sorting defects were performed using electron micrographs of ultrathin sections and analyzed using NIH Image J version 1.47 software (http://rsb.info.nih.gov/ij/). An entire sciatic nerve cross-section per animal was reconstructed by merging several high magnification photographs taken by bright field microscopy at ×100 magnification. The number of myelinated axons per nerve were analyzed in ultrathin sections using a JEOL 1200 EXII electron microscope.

### Transient transfections in rat SCs

For plasmid transfections, rat SCs were transfected with expression vectors using Lipofectamine 3000 (Life Technologies, L3000008) per the manufacturer’s protocol and assayed for immunocytochemistry or qPCR analysis. For siRNA knockdown in SCs, we used Lipofectamine RNAiMAX (Life Technologies, 13778075) per the manufacturer’s instructions. SCs were induced to differentiate after 24 h of transfection for 9 h before harvesting for immunocytochemistry or qPCR analysis. For siRNA knockdown, target sequence for CTCF siRNA is as follows: SASI_Rn01_00100578, SASI_Rn01_00100579, SASI_Rn01_00100580, SASI_Rn01_00100581. control siRNA: MISSION siRNA Universal Negative Control #1 (SIC001).

### Western blotting and co-immunoprecipitation

For western blotting, cells were lysed in modified RIPA buffer (50 mM Na-Tris, pH 7.4, 150 mM NaCl, 1% (v/v) NP-40, 0.25% sodium deoxycholate, 1 mM dithiothreitol, 10 mM NaF, 1 mM active sodium vanadate, 1 mM PMSF and 1× a cocktail of cOmplete protease inhibitors (Roche Applied Science) and centrifuged at 15,000 × *g*. for 15 min at 4 °C. After the determination of protein concentration (Bio-Rad), the lysates were separated by 4–12% SDS-PAGE. We used antibodies against CTCF (rabbit, Cell Signaling Technology, 3417 S, 1:1000), MBP (goat, Santa Cruz Biotechnology, sc-13914, 1:500), MPZ (rabbit, Abcam, ab31851, 1:1000), KROX20/EGR2 (rabbit, Santa Cruz Biotechnology, sc-20690, 1:200 or Guinea pig, provided by Michael Wegner, 1:1000), SUZ12 (rabbit, Cell Signaling Technology, 3737 S, 1:1000), EED (rabbit, Millipore, 17-10034, 1:1000), EZH2 (rabbit, Cell Signaling Technology, 5246 P, 1:1000), H3 (rabbit, Cell Signaling Technology, 4499 S, 1:1000), H3K27me3 (rabbit, Cell Signaling Technology, 9733 S, 1:1000), H3K27me2/3 (mouse, Active motif, 39536, 1:1000), H3K36me3 (rabbit, Abcam, ab9050, 1:1000), H3K4me1 (mouse, Active motif, 39635, 1:1000), H3K27ac (rabbit, Cell Signaling Technology, 4353 S, 1:1000), and GAPDH (mouse, Millipore, MAB374, 1:5000). Bands were visualized with secondary antibodies conjugated to horseradish peroxidase (Jackson ImmunoResearch Laboratories, 111-035-144, 705-035-147, and 115-035-062) and ECL western blotting detection reagents (Pierce) per the manufacturer’s instructions.

For immunoprecipitation, HEK293T cells cultured in 10% FBS/DMEM were transfected with expression vectors using PolyJet (Signagen) for 48 h. HEK293T or differentiating rat Schwann cells were lysed in NP-40 buffer (170 mM NaCl, 10 mM EDTA, 50 mM Tris pH 7.4, 50 mM NaF and 0.5% NP-40) supplemented with protease inhibitor cocktail and phosphatase inhibitors (Roche Diagnostics Inc.). A total of 300 μg of cell lysate proteins were incubated with 2 μg IgG or appropriate antibodies and immunoprecipitated using Protein A/G beads (Santa Cruz Biotechnology, sc-2003). Western blotting was performed using chemiluminescence with the ECL kit (Pierce). The antibodies used were CTCF (rabbit, Cell Signaling, 3417 S, 1:100), SUZ12 (rabbit, Cell Signaling Technology, 3737 S, 1:1000 for Western), anti-Flag (rabbit, 14793 S or mouse, 8146 S, Cell Signaling Technology, 1:200), and HA-tag (mouse, Cell Signaling, 2367 S, 1:200). Secondary antibodies conjugated to HRP were from Jackson ImmunoResearch Laboratories (111-035-144, and 115-035-062, 1:5000).

### Tamoxifen injection for *Ctcf* inducible knockout

We used Tamoxifen (Sigma, T5648) dissolved to a stock concentration of 20 mg ml^−1^ in a vehicle of ethanol and sunflower seed oil (1:9 v/v). For perinatal tamoxifen injections, tamoxifen stock was injected i.p. at 20 mg ml^−1^ for 5 consecutive days to lactating mothers, thus administering tamoxifen to pups, beginning at P0. Sciatic nerves of pups were analyzed at P14 for either immunostaining or electron microscopy. For adult tamoxifen treatment, 100 μl (20 mg ml^−1^) was administered by i.p. injection once daily for 5 consecutive days. Mice were treated again for 5 days after a 2-day rest period. Control mice were treated identically.

### Sciatic nerve transection injury

Mice at 6–8 weeks were under general anesthesia with injection of a mixture of ketamine (90 mg kg^−1^ body weight) and xylazine (10 mg kg^−1^ body weight). Right sciatic nerves were exposed and transected at midthigh level. Exposed left sciatic nerves were used as uncut controls. Mice were treated with tamoxifen to delete floxed *Ctcf* alleles following injury. Nerves were collected at day 14 or 56 following surgery and processed for immunohistochemistry or EM.

### RNA isolation and real-time RT-PCR analysis

RNAs were isolated with the RNeasy Plus Mini kit (Qiagen, 74104) from cultured cells or snap-frozen sciatic nerves. cDNA was synthesized from 1 μg RNA using iScript Reverse Transcription Supermix (BioRad) according to the manufacturer’s instructions. qRT-PCR was performed using the StepOnePlus Real-Time PCR System (Applied Biosystems inc). qRT-PCR was performed using quantitative SYBR green PCR mix (BioRad). PCR primer sequences are available upon request. qRT-PCR analysis is based on the ΔΔCT method with normalization of the raw data to GAPDH genes according to the previous method^64^. For each gene, ΔCT was calculated by subtracting CT_GAPDH_ from CT_GENE_ in either the control or experimental group. We set the average ΔCT of the control as a calibrator, then the 2^–ΔΔCT^ method was used to calculate each relative expression in both control or experimental groups. The values in the control were normalized to 1 by dividing each data point with the averaged control value.

### DRG explant cultures

DRG neurons were isolated from embryonic day (E) 16.5 rat spinal cords and plated as explants on collagen-coated coverslips. Cultures were maintained in serum-free neurobasal medium (NB medium; 2% B27 supplement, 2 mM L-glutamine, 0.4% glucose, and 50 ng/ml 2.5 S nerve growth factor (NGF) (Harlan, 005017). Non-neuronal cells were removed via feeding the cultures with NB medium containing 5-fluorodeoxyuridine and uridine. SCs were isolated from sciatic nerves taken at P2 and expanded in SC proliferation medium. SC–DRG co-cultures were established through seeding purified DRG neuron cultures with 100,000 SCs in culture medium (MEM, 10% FBS, 2 mM L-glutamine, 0.4% glucose, and 50 ng/ml 2.5 S NGF). 3 days after SC plating, SC medium was supplemented with 50 μg ml^−1^ ascorbic acid (Sigma, A0278) to initiate myelination. SC–DRG co-cultures were allowed to myelinate for 10 days, with fresh media provided every 2 days. To determine the extent of myelination in SC–DRG co-cultures, the total number of MBP^+^ segments was counted in micrographs from 10–12 random fields per coverslip.

### RNA-seq and data analysis

RNA from siControl and si*Ctcf* SCs were extracted using TRIZOL (Life Technologies) followed by purification using a RNeasy Mini Kit (Qiagen). RNA-seq libraries were prepared using the Illumina TruSeq RNA Library Prep Kit v2 and sequenced by a HiSeq 2500 sequencer. RNA-seq reads were aligned to mm10 using TopHat 2.1.1 with default settings (http://ccb.jhu.edu/software/tophat/index.shtml). We used Cuffdiff2 2.2.1 to (1) estimate fragments per kilobase of transcript per million mapped reads (FPKM) values for known transcripts and to (2) analyze differentially expressed transcripts. In all differential expression tests, a difference was considered significant if the *p*-value was less than 0.05 and fold-change more 1.5 (Cuff-diff default). DEseq were used for RNA-seq of control and *Ctcf* cKO sciatic nerves from P7. Statistical analyses were performed to identify differentially expressed genes for each comparison using the negative-binomial model of read counts as implemented in the Bioconductor DESeq 1.39.0 package (https://bioconductor.org/packages/release/bioc/html/DESeq.html). Gene ontology (GO) analysis was performed using ToppGene Suite (https://toppgene.cchmc.org/) and Gene Set Enrichment Analysis (GSEA 4.0.1; http://www.broadinstitute.org/gsea/index.jsp).

### Chromatin immunoprecipitation sequencing (ChIP-seq) assays

ChIP assays were performed with minor modifications^43^. Purified rat SCs were fixed for 15 min at room temperature with 1% formaldehyde-containing medium. Nuclei were isolated and sonicated in sonication buffer (10 mM Tris-HCl pH 8.0, 1 mM EDTA, 0.5 mM EGTA and protease inhibitor cocktail). Sonicated chromatin (~300 µg) was used for immunoprecipitation by incubation with appropriate antibodies (4 μg) overnight at 4 °C. Prerinsed magnetic protein A/G beads (50 μL, Thermo Fisher Scientific, 26162) were added to each ChIP reaction and reactions were incubated for 1 h at 4 °C. The beads were then incubated in 200 ml elution buffer at 65 °C for 20 min to elute immunoprecipitated materials. The ChIP-seq libraries were prepared using NEBNext ChIP-seq Library Prep Master Mix Set for Illumina (NEB catalogue number E6240L) and then run on the Illumina sequencer HiSeq 2500. We used antibodies CTCF (rabbit, Cell Signaling, #3418), H3K27Ac (rabbit, Active motif, 39135), or H3K27me3 (rabbit, Cell Signaling, 9733 s) for ChIP. The crosslinked and sonicated chromatins without immunoprecipitation were used as input controls. For ChIP, real-time PCR was performed using quantitative SYBR green PCR mix (Bio-Rad, 1725121). The values of IgG were normalized to 1.

### ChIP-seq peak-calling and data analysis

Reads of ChIP-seq data were aligned to Rn5 using Bowtie2 v2.3.5.1with the following options: -p 8, -m 1 (http://bowtie-bio.sourceforge.net). Peak calling was performed using MACS version 1.4.2 (Model-based Analysis of ChIP-seq) (http://liulab.dfci.harvard.edu/MACS) with a *p*-value cutoff of 10^−9^ and compared the peak sets using the ENCODE (v90) overlap rules. Motifs were predicted using the HOMER v4.11 program (http://homer.salk.edu/homer). The heatmaps were drawn using the Heatmap tools provided by Cistrome (http://cistrome.org/ap). Tracks were shown in Mochiview version 1.46. We used the FastQC 0.11.9 pipeline (http://www.bioinformatics.babraham.ac.uk/projects/fastqc/), a quality control tool for high-throughput sequence data, to assure the quality of raw sequence data from ChIP-seq and ATAC-seq. Mapping tags, parameters of alignments, peak enrichment information, and FastQC results for the ChIP-seq and ATAC-seq dataset were included in Supplementary Data 4.

### Assay for transposase-accessible chromatin using sequencing (ATAC-Seq)

We isolated nuclei of ~50,000 cells from a pool of control or *Ctcf*-knockdown SCs from two independent experiments in a cold lysis buffer (10 mM Tris-HCl, pH 7.4, 10 mM NaCl, 3 mM MgCl_2_, 0.1% IGEPAL CA-630). After spinning down at 500 × *g* for 10 min at 4 C, nuclei were resuspended in transposition mix containing 2 × reaction buffer (TD, Illumina, 20034197), Nextera Tn5 Transposase (TDE1, Illumina, 15027865) at 37 °C for 30 min. Immediately following transposition, DNA were purified using a Qiagen MinElute PCR Purification Kit. Transposed DNA fragments were subsequently amplified and the amplified library was purified using Qiagen MinElute PCR Purification Kit. Libraries were generated using the Ad1_noMX and barcoded primers and were amplified for 11 total cycles. Libraries were purified with AMPure beads (Beckman Coulter, A63880) to remove contaminating primer dimers. All libraries were sequenced on the Illumina HiSeq 2500 with 75 bp single-end reads.

Reads of ATAC-seq data were aligned to rn5 genome using Bowtie2 v2.3.5.1 with the following options: –best–chunkmbs 200 (http://bowtie-bio.sourceforge.net). Peak calling and identification were performed using Model-based analysis of MACS version 1.4.2 (https://github.com/taoliu/MACS) with specific parameters without the prebuilt model: –shift 75 –extsize 150 –nomodel –call-summits –nolambda –keep-dup all −p 0.01, to call peaks, which extend and shift the fragments to get the region cut by the Tn5 sites. The read density profiles were normalized by the library size. Tracks were shown in Mochiview version 1.46. We calculated the peak_RPKM, then used GSEA (4.0.1) to analyze the enrichment of signature gene sets in siControl and si*Ctcf* cells^65^. Mapping tags, parameters of alignments, peak enrichment information, and FastQC 0.11.9 quality control results for the ATAC-seq dataset was included in Supplementary Data 4.

### Chromosome conformation capture (3C) assays

siControl or si*Ctcf* rat SCs (2 × 10^6^) were differentiated for 9 h and 1 × 10^7^ cells were crosslinked with 2% formaldehyde 10% FCS/PBS at room temperature for 10 min, followed by 1 M glycine quenching, pellet was lysed in a cold lysis buffer [10 mM Tris-HCl, pH 7.5; 10 mM NaCl; 5 mM MgCl_2_; 0.1 mM EGTA; 1× complete protease inhibitor (Roche, 11836145001)]. Then nuclei were digested with HindIII (NEB R3104s) overnight at 37 °C, and then T4 ligation (NEB M0202S) at 16 °C for 4 h. Then we use phenol-chloroform to purify the DNA pellet. 3C ligation products were quantified in triplicates by quantitative SYBR Green real-time PCR^66,67^. The relative interaction 3C signals were calculated according to a locus-specific BAC standard curve and to 3C signals and normalized to the level of the *Gapdh* locus. We chose the probe primer sequences for 3C experiments according to the potential enhancer elements marked by the activating histone mark H3K27ac in SCs.

### Statistical analysis

All analyses were done using Microsoft Excel version 16.16.23 or GraphPad Prism 6.00 (San Diego California, http://www.graphpad.com). Data are shown in dot plots or bar graphs as mean ± SEM; *P* < 0.05 is deemed statistically significant. Data distribution was assumed to be normal, but this was not formally tested. Count data were assumed to be nonparametric, and appropriate statistical tests were used. Statistical analysis was performed using two-tailed or one-tailed unpaired Student’s *t*-tests between two samples and one-way ANOVA with Tukey’s post-hoc analysis for multiple comparisons, log-rank test for survivals or as indicated. Permutation test was carried out based on the paired *t*-test statistic and 100,000 permutations. Quantifications were performed from at least three experimental groups in a blinded fashion. The n value was defined as the number of experiments that were repeated independently with similar results. No statistical methods were used to predetermine sample sizes, but our sample sizes are similar to those generally employed in the field. Samples from the genotyped animals were randomly assigned for experimental analysis and data collection, and data were quantified with blinding.

### Reporting summary

Further information on research design is available in the Nature Research Life Sciences Reporting Summary linked to this article.

## Supplementary information

Supplementary Information

Description of Additional Supplementary Information

Supplementary Data 1

Supplementary Data 2

Supplementary Data 3

Supplementary Data 4

Reporting Summary

## Data Availability

All high-throughput data generated in the paper are deposited in the NCBI Gene Expression Omnibus (GEO). The accession numbers are GSE138117. ChIP-seq datasets for H3K27me3 and p300 are extracted from GSE84265 and GSE93161, respectively. Egr2^Lo^ decreased and increased genes were obtained from https://www.pnas.org/content/suppl/2005/02/03/0407836102.DC1#F5 (Supporting Table 2). The list of differentially regulated genes between the *Eed* cKO and WT nerves were obtained from https://onlinelibrary.wiley.com/doi/full/10.1002/glia.23500 (Supporting Information Table 2). The data supporting this study are available in the Article, Supplementary Information, Source Data or available from the corresponding authors upon reasonable requests. A reporting summary for this Article is available as a Supplementary Information file. Source data are provided with this paper.

## Source data

Source Data
