# Supplementary material for: CTCF-mediated chromatin looping in EGR2 regulation and SUZ12 recruitment critical for peripheral myelination and repair
Source: Nat Commun. 2020 Aug 17;11:4133. doi: 10.1038/s41467-020-17955-2 (PMC7431862; doi:10.1038/s41467-020-17955-2)
Supplement: Supplementary file 1 — Supplementary Information [file 41467_2020_17955_MOESM1_ESM.pdf]

Supplementary information

CTCF-mediated chromatin looping in EGR2 regulation and SUZ12 recruitment critical for peripheral myelination and repair

Wang et al.

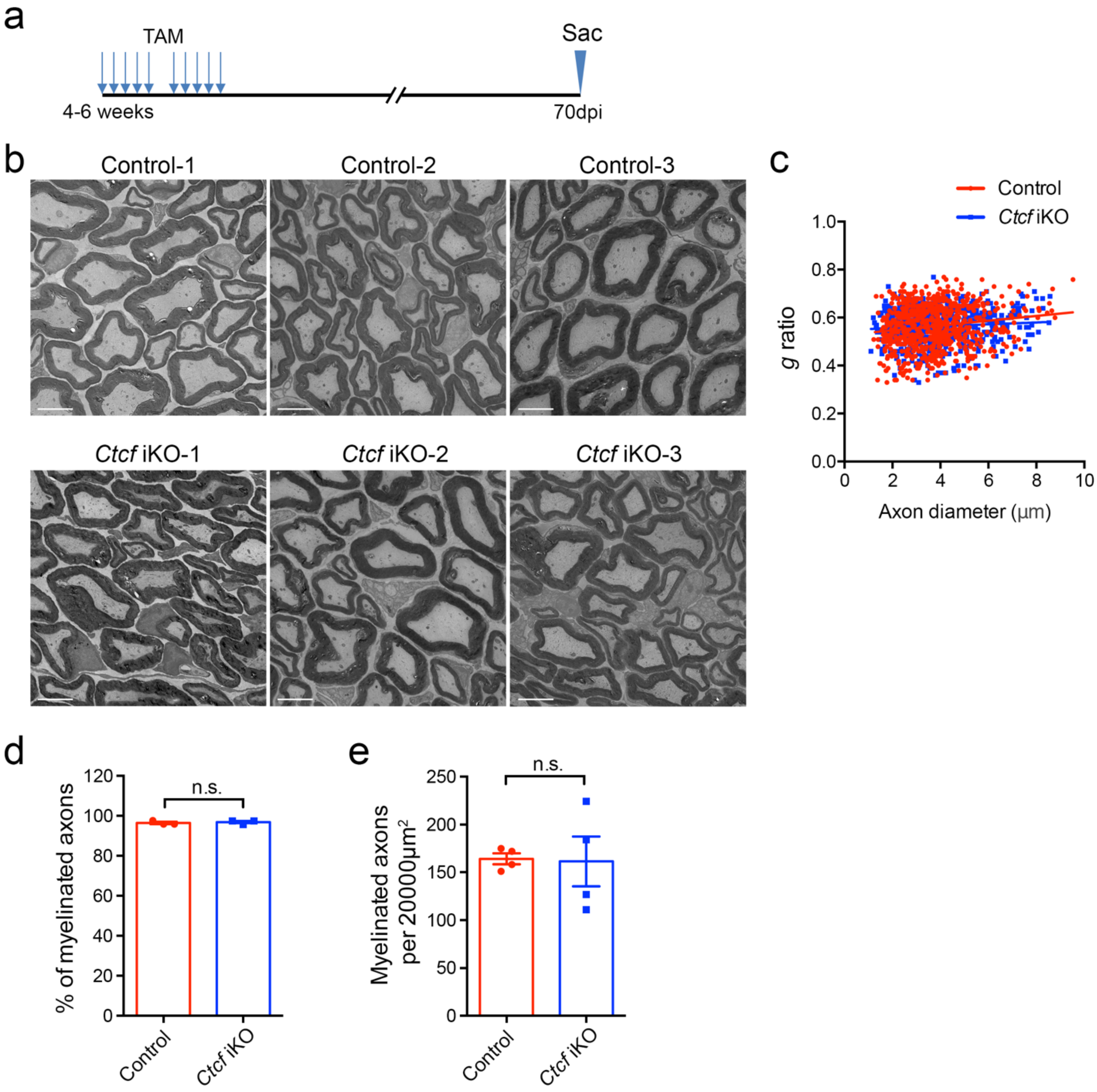

Supplementary Fig. 1. CTCF is not required for myelin maintenance in adult peripheral nerves

(**a**) A diagram showing the tamoxifen (TAM) administration scheme. Beginning at ~6 weeks of age, mice were treated with TAM via i.p. injection for 5 days with a 2-day break, and then treat again for 5 days. The nerves were analyzed at 70 days after the last injection.

(**b**) Representative EM images of cross sections of sciatic nerves from adult control and *Ctcf* iKO mice. n = 3 animals/genotype. Scale bar: 6 µm.

(**c**) Quantification of *g* ratios of axons from control and *Ctcf* iKO mice. *n* > 500 axons from 3 control and 3 *Ctcf* iKO mice; *P* = 0.98.

(**d**) Percentage of the myelinated axons sciatic nerves from adult control and *Ctcf* iKO mice. n.s., no significance, $n = 3$ animals/genotype, $P = 0.66$.

(**e**) Quantification of myelinated axon numbers per 2000 μm^2 sections of sciatic nerves from adult control and *Ctcf* iKO mice. n.s., no significance, $n = 4$ animals/genotype, $P = 0.93$.

Data are presented as means ± SEM., two-tailed unpaired Student's t-test.

Source data are provided as a Source Data file.

a

Genes	Control-1	Control-2	*Ctcf* cKO-1	*Ctcf* cKO-2	Fold change	Adjusted P-value
Nfatc3	9.79	10.28	12.56	12.50	1.07	0.456
Nfatc4	3.28	3.45	3.19	4.29	0.95	0.876
Yap1	27.41	29.33	35.73	35.76	1.08	0.509
Tead1	52.86	48.62	50.15	50.07	0.85	0.126
*Tead2**	11.89	12.31	26.21	26.65	1.88	0.000
*Tead3**	12.55	12.29	6.24	6.28	0.43	0.000
*Tead4**	4.49	3.68	1.60	1.22	0.30	0.000

* FC > 1.5, and *P* < 0.5

b

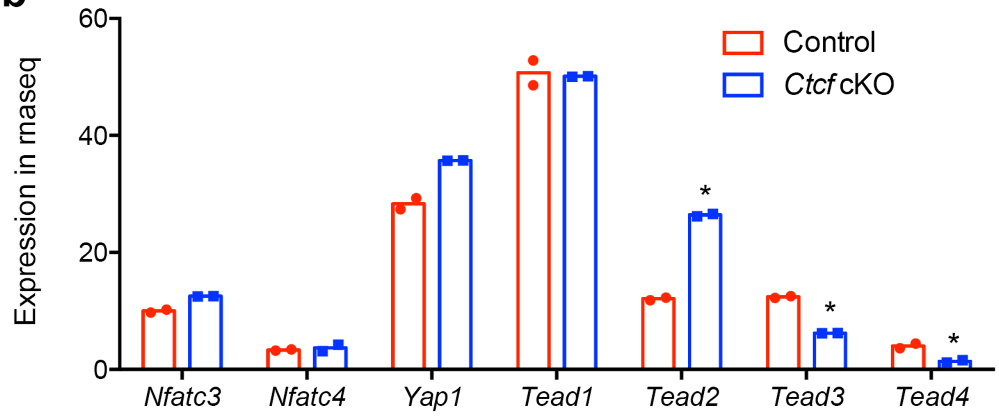

Supplementary Fig. 2. Expression of potential *Egr2* activating factors in *Ctcf* cKO sciatic nerves. (**a,b**) Expression values (FPKM) (**a**) or graph (**b**) of *Nfatc3/4*, *Yap1*, and *Tead1-4* genes from RNA-seq data in Control and *Ctcf* cKO sciatic nerves (n = 2 animals/genotype). *, Fold change >1.5, $P < 0.05$, differential gene expression and statistical analysis were performed by the DEseq package.

a

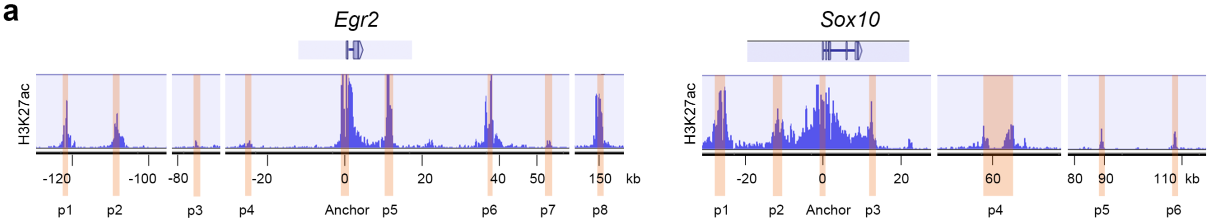

b

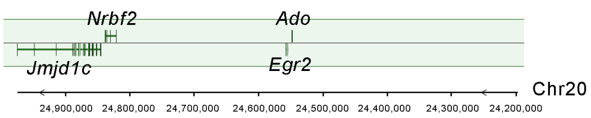

c

Ctcf cKO compared to Control

Gene	Ctrl1	Ctrl2	cKO1	cKO2	Fold change	adjustd p-value
Ado	45.1	43.3	39.3	39.8	1.30	0.003
*Egr2**	67.1	62.2	31.5	30.2	2.44	4.84E-24
Nrbf2	7.6	7.9	11.7	14.0	1.42	0.053
Jmjd1c	7.5	8.0	10.2	9.9	1.11	0.334

*: FC > 1.5 and *P* < 0.05

d

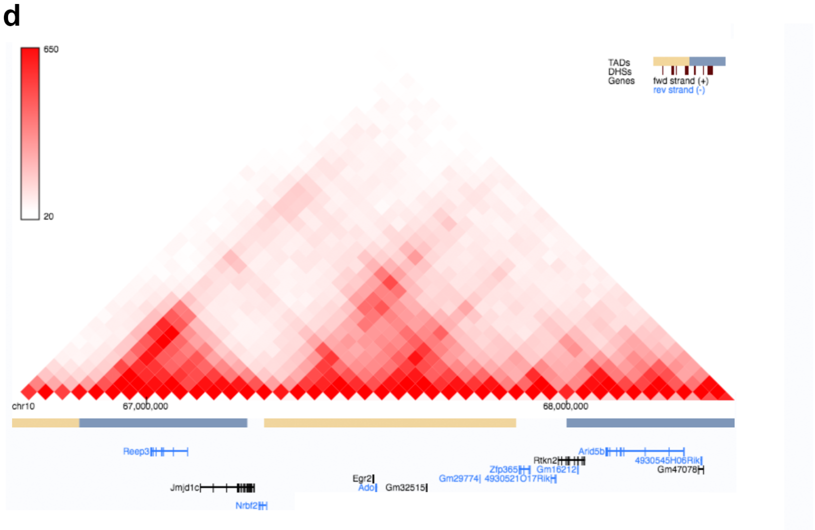

Supplementary Fig. 3. Neighboring genes around *Egr2* are not affected by CTCF depletion.

(**a**) Schematic of the *Egr2 and Sox10* indicating the positions of the primers used in the 3C assays.

(**b**) Genome browser showing the locus of *Egr2* and neighboring genes.

(**c**) Gene expression of *Egr2*, *Ado*, *Nrbf2*, and *Jmjd1c* genes among RNA-seq data in Control and *Ctcf* cKO sciatic nerves. *, Fold change >1.5, $P < 0.05$, differential gene expression and statistical analysis were performed by DEseq package.

(**d**) Snapshots of 1.72 Mb of Hi-C data at 40 kb resolution around *Egr2* gene loci in mouse neural progenitors (NPCs) derived from embryonic stem cells (mESCs) (http://promoter.bx.psu.edu/hi-c/view.php). The rectangle bars represent the topologically associating domains (TAD).

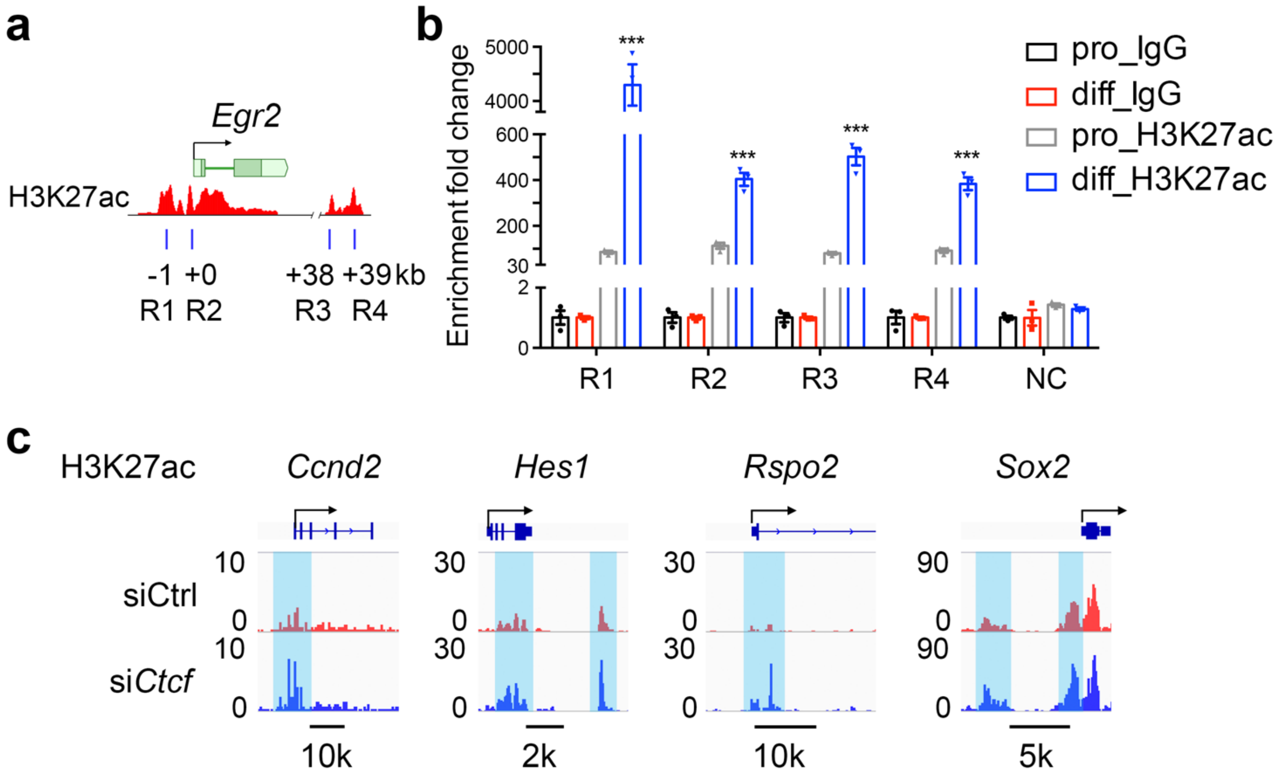

Supplementary Fig. 4. H3K27ac activity around *Egr2* and differentiation inhibitory gene loci

(**a,b**) Diagram of H3K27ac activity around the *Egr2* locus based on H3K27ac ChIP-seq profiles in differentiating SCs (**a**). ChIP-qPCR assay for H3K27ac enrichment in the promoter and enhancer elements of the *Egr2* locus in proliferative and differentiated SCs (**b**). Data are presented as means ± SEM; The values of IgG were normalized to 1. $n = 3$ independent experiments; $^{***}P < 0.01$; two-tailed unpaired Student's t test. R1-4, primer locations; NC, non-target genomic region control, $P_{(R1)} = 0.00037$, $P_{(R2)} = 0.00076$, $P_{(R3)} = 0.00039$, $P_{(R4)} = 0.00057$, $P_{(NC)} = 0.17$.

(**c**) Representative ChIP-seq tracks showing H3K27ac signals on SC differentiation inhibitory gene loci in control and si*Ctcf* SCs. n = 1 in each condition with 20 million cells.

Source data are provided as a Source Data file.

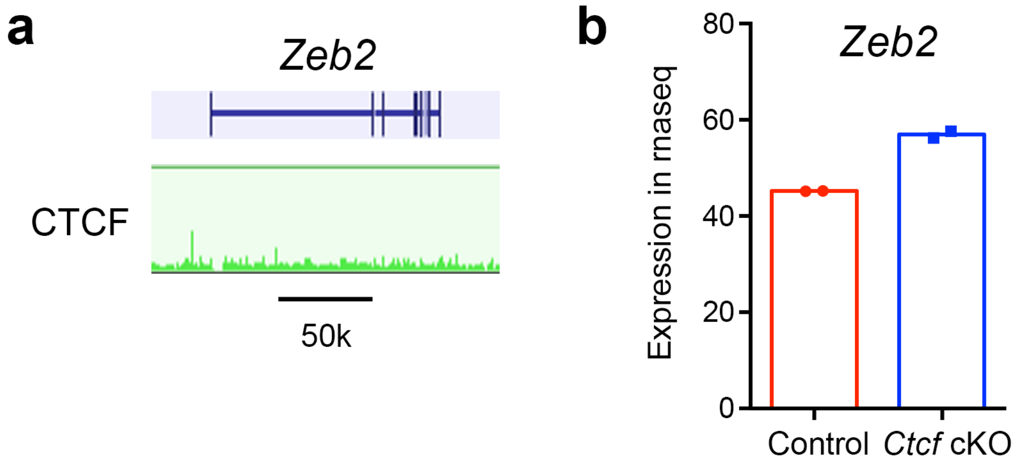

Supplementary Fig. 5. Expression of SC regulatory genes in *Ctcf* cKO sciatic nerves

(**a**) Genome browser tracks over *Zeb2* loci with ChIP-seq density mapping of CTCF from SCs.

(**b**) Expression of *Zeb2* gene among RNA-seq data in control and *Ctcf* cKO sciatic nerves (n = 2 animals/genotype).

Source data are provided as a Source Data file.
