# Supplementary material for: CTCF-mediated chromatin looping in EGR2 regulation and SUZ12 recruitment critical for peripheral myelination and repair
Source: Nat Commun. 2020 Aug 17;11:4133. doi: 10.1038/s41467-020-17955-2 (PMC7431862; doi:10.1038/s41467-020-17955-2)
Supplement: Supplementary file 2 — Description of Additional Supplementary Information [file 41467_2020_17955_MOESM2_ESM.pdf]

Description of Additional Supplementary Files

File Name: Supplementary Data 1
Description: Gene expression with differential chromatin accessibility

File Name: Supplementary Data 2
Description: Details of CTCF ChIP-seq peaks overlapped with either H3K27me3 or H3K27ac ChIP-seq peaks

File Name: Supplementary Data 3
Description: Upregulated genes in si*Ctcf* SCs targeted by CTCF

File Name: Supplementary Data 4
Description: QC and sequence information for ATAC-seq and ChIP-seq
