# Supplementary material for: CTCF-mediated chromatin looping in EGR2 regulation and SUZ12 recruitment critical for peripheral myelination and repair
Source: Nat Commun. 2020 Aug 17;11:4133. doi: 10.1038/s41467-020-17955-2 (PMC7431862; doi:10.1038/s41467-020-17955-2)
Supplement: Supplementary file 7 — Reporting Summary [file 41467_2020_17955_MOESM7_ESM.pdf]

nature research

Corresponding author(s): Qing Richard Lu

Last updated by author(s): Jun 24, 2020

Reporting Summary

Nature Research wishes to improve the reproducibility of the work that we publish. This form provides structure for consistency and transparency in reporting. For further information on Nature Research policies, see our Editorial Policies and the Editorial Policy Checklist.

Statistics

For all statistical analyses, confirm that the following items are present in the figure legend, table legend, main text, or Methods section.

n/a	Confirmed	
☐	☒	The exact sample size (*n*) for each experimental group/condition, given as a discrete number and unit of measurement
☐	☒	A statement on whether measurements were taken from distinct samples or whether the same sample was measured repeatedly
☐	☒	The statistical test(s) used AND whether they are one- or two-sided<br>*Only common tests should be described solely by name; describe more complex techniques in the Methods section.*
☐	☒	A description of all covariates tested
☐	☒	A description of any assumptions or corrections, such as tests of normality and adjustment for multiple comparisons
☐	☒	A full description of the statistical parameters including central tendency (e.g. means) or other basic estimates (e.g. regression coefficient) AND variation (e.g. standard deviation) or associated estimates of uncertainty (e.g. confidence intervals)
☐	☒	For null hypothesis testing, the test statistic (e.g. *F*, *t*, *r*) with confidence intervals, effect sizes, degrees of freedom and *P* value noted<br>*Give P values as exact values whenever suitable.*
☒	☐	For Bayesian analysis, information on the choice of priors and Markov chain Monte Carlo settings
☒	☐	For hierarchical and complex designs, identification of the appropriate level for tests and full reporting of outcomes
☒	☐	Estimates of effect sizes (e.g. Cohen's *d*, Pearson's *r*), indicating how they were calculated

Our web collection on statistics for biologists contains articles on many of the points above.

Software and code

Policy information about availability of computer code

Data collection	No software was used during data collection.<br>RNA-seq libraries were prepared using Illumina RNA-Seq Preparation Kit and sequenced by a HiSeq 2500 sequencer.<br>The ChIP-seq libraries were prepared using NEBNext ChIP-seq Library Prep Master Mix Set for Illumina (NEB catalogue number E6240L) and then run on the Illumina sequencer HiSeq 2500.<br>ATAC-seq libraries were purified with AMPure beads (Agencourt) to remove contaminating primer dimers. All libraries were sequenced on the Illumina HiSeq 2500 with 75 bp single-end reads.
Data analysis	RNA-seq: Reads mapped to mm10 or rn5 whole genome by Tophat 2.1.1 (http://ccb.jhu.edu/software/tophat/index.shtml). We used Cuffdiff2 2.2.1 and Bioconductor DESeq (1.39.0) package to identify differentially expressed genes. Gene ontology (GO) analysis was performed using ToppGene Suite (https://toppgene.cchmc.org/) and Gene Set Enrichment Analysis (GSEA 4.0.1; http://www.broadinstitute.org/gsea/index.jsp).<br>ChIP-seq: Reads mapped to rn5 whole genome by Bowtie2v2.3.5.1 with default options. Peak calling was performed using MACS1.4.2 with a p value cutoff of 10-9 and compared the peak sets using the ENCODE(v90) overlap rules. Motif was analyzed using HOMERv4.11 (http://homer.salk.edu/homer/index.html);<br>ATAC-seq: Reads mapped to rn5 whole genome by Bowtie2v2.3.5.1 with default options. Peak calling was performed using Model-based analysis of MACS version 1.4.2 with special parameter: --shift -75 --extsize 150 --nomodel --call-summits --nolambda --keep-dup all -p 0.01, to call peak, which extend and shift the fragments to get the region cut by the Tn5 sites.<br>Others: Mochiview version1.46; Cistrome (http://cistrome.org/ap). Prism 6.0; Image J 1.47; Microsoft Excel 16.16.23.

For manuscripts utilizing custom algorithms or software that are central to the research but not yet described in published literature, software must be made available to editors and reviewers. We strongly encourage code deposition in a community repository (e.g. GitHub). See the Nature Research guidelines for submitting code & software for further information.

Data

Policy information about availability of data

All manuscripts must include a data availability statement. This statement should provide the following information, where applicable:

- Accession codes, unique identifiers, or web links for publicly available datasets
- A list of figures that have associated raw data
- A description of any restrictions on data availability

Data availability
All high-throughput data generated in the paper are deposited in the NCBI Gene Expression Omnibus (GEO). The accession numbers are GSE138117[https://www.ncbi.nlm.nih.gov/geo/query/acc.cgi?acc=GSE138117]. ChIP-seq datasets for H3K27me3 and p300 are extracted from GSE84265[https://www.ncbi.nlm.nih.gov/geo/query/acc.cgi?acc=GSE84265] and GSE93161[https://www.ncbi.nlm.nih.gov/geo/query/acc.cgi?acc=GSE93161], respectively.
Egr2Lo decreased and increased genes were obtained from https://www.pnas.org/content/suppl/2005/02/03/0407836102.DC1#F5 (Supporting Table 2).
The list of differentially regulated genes between the Eed cKO and WT nerves were obtained from https://onlinelibrary.wiley.com/doi/full/10.1002/glia.23500 (Supporting Information Table 2).
The source data underlying Figs 1a, b, d, e, 2a, d, e, g–i, 3c, g, m, 4a, b, d–f, h, j, l, m, o, 5d, f, h, j, 6e, 7e, 8e–k and 9c, m–o, 10d, e and Supplementary Figs 1c–e, 4b, and 5b are provided as a Source Data file. The data supporting this study are available in the Article, Supplementary Information, Source Data or available from the authors upon reasonable requests. Source data are provided with this paper.

Field-specific reporting

Please select the one below that is the best fit for your research. If you are not sure, read the appropriate sections before making your selection.

- [x] Life sciences
- [ ] Behavioural & social sciences
- [ ] Ecological, evolutionary & environmental sciences

For a reference copy of the document with all sections, see nature.com/documents/nr-reporting-summary-flat.pdf

Life sciences study design

All studies must disclose on these points even when the disclosure is negative.

Sample size	Sample sizes were indicated in the legend of each Figure and Supplementary Figure. No statistical methods were used to predetermine sample sizes.<br>The sample sizes for western bloting were at least 2 for each group.<br>The sample sizes for RNA-seq and ATAC-seq were 2 for each group.<br>The sample sizes for ChIP-seq were 1 for each group.<br>The sample sizes for others were at least 3 for each group.<br>Our sample sizes are similar to those generally employed in the life sciences field.<br>(Soleilhavoup, C., Travaglio, M., Patrick, K. et al, 2020. https://doi.org/10.1038/s41467-020-16947-6<br>Guo, H., Ci, X., Ahmed, M. et al, 2019. https://doi.org/10.1038/s41467-018-08133-6<br>Maas, D.A., Eijsink, V.D., Spoelder, M. et al, 2020. https://doi.org/10.1038/s41467-020-16218-4<br>He, X., Zhang, L., Queme, L. et al, 2018. https://doi.org/10.1038/nm.4483<br>Li, J., Huang, K., Hu, G. et al, 2019. https://doi.org/10.1038/s41467-019-08949-w)
Data exclusions	No data were excluded from analyses.
Replication	The experimental findings were reliably reproduced, for representative data used for statistical analysis, the number of animals or experiments is described in corresponding figure legends.
Randomization	Samples from the genotyped animals were randomly assigned for experimental analysis and data collection
Blinding	For cell-based experiments, EM, immunohistochemistry, the genotypes of cells/animals were known before the conduction of experiments. For image analysis, cell and EM images were quantified in a blinded manner.

Reporting for specific materials, systems and methods

We require information from authors about some types of materials, experimental systems and methods used in many studies. Here, indicate whether each material, system or method listed is relevant to your study. If you are not sure if a list item applies to your research, read the appropriate section before selecting a response.

Materials & experimental systems

n/a	Involved in the study
☐	☒ Antibodies
☐	☒ Eukaryotic cell lines
☒	☐ Palaeontology and archaeology
☐	☒ Animals and other organisms
☒	☐ Human research participants
☒	☐ Clinical data
☒	☐ Dual use research of concern

Methods

n/a	Involved in the study
☐	☒ ChIP-seq
☒	☐ Flow cytometry
☒	☐ MRI-based neuroimaging

Antibodies

Antibodies used

For western blot experiments, we used antibodies against CTCF (rabbit, Cell Signaling Technology, 3417S), MBP (goat; Santa Cruz Biotechnology, sc-13914), MPZ (rabbit; Abcam, ab31851), EGR2 (rabbit, Santa Cruz Biotechnology, sc-20690), SUZ12 (rabbit, Cell Signaling Technology, 3737S), EED (rabbit, Millipore, 17-10034), EZH2 (rabbit, Cell Signaling Technology, 5246P), H3 (rabbit, Cell Signaling Technology, 4499S), H3K27me3 (rabbit, Cell Signaling Technology, 9733S), H3K27me2/3 (mouse, Active motif, 39536), H3K36me3 (rabbit, Abcam, ab9050), H3K4me1 (mouse, Active motif, 39635), H3K27ac (rabbit, Cell Signaling Technology, 4353S), and GAPDH (mouse, Millipore, MAB374). Secondary antibodies conjugated to horseradish peroxidase (Jackson ImmunoResearch Laboratories, 111-035-144, 705-035-147, and 115-035-062) were used.

For immunoprecipitation experiments, the antibodies used were anti-CTCF (rabbit; Cell Signaling, 3417S), anti-SUZ12 (rabbit, Cell Signaling Technology, 3737S), anti-Flag (rabbit, 14793S or mouse, 8146S, Cell Signaling Technology) and HA-tag (Mouse, Cell Signaling Technology, 2367S). Secondary antibodies conjugated to HRP were from Jackson ImmunoResearch Laboratories (111-035-144, and 115-035-062)

For immunofluorescence experiments, we used antibodies to CTCF (rabbit; Cell Signaling, #3418), SOX10 (goat, Santa Cruz Biotechnology, sc-17342; rabbit, Abcam; ab155279), Oct6 (goat; Santa Cruz Biotechnology, sc-11661), EGR2 (rabbit, Santa Cruz Biotechnology, sc-20690), MBP (goat; Santa Cruz Biotechnology, sc-13914), SOX2 (goat; Santa Cruz Biotechnology, sc-17320), Ki67 (rabbit; Thermo Scientific, RM-9106-S0), BrdU (rat; Abcam, ab6326), cleaved caspase 3 (rabbit; Cell Signaling, #9661), NF-M (Rabbit, Millipore, AB1987). Secondary antibodies conjugated to Cy2, Cy3 or Cy5 were from Jackson ImmunoResearch Laboratories catalog numbers 705-165-147, 705-225-147, 711-225-152, 711-165-152, 711-175-152, 715-165-150 and 712-165-150.

For ChIP experiments, we used antibodies CTCF (rabbit; Cell Signaling, #3418), H3K27Ac (Active motif, 39135), and H3K27me3 (rabbit, Cell Signaling Technology, 9733S).

Antibodies are also listed in the Methods section under their respective experimental method.
We choose these antibodies by the product data sheets, literature and our pilot studies.

Validation

Primary antibodies were validated as follows:
- Anti-CTCF (rabbit, Cell Signaling Technology, 3417S): validated by the manufacturer "Application: Western Blotting, Immunoprecipitation. Specificity / Sensitivity: CTCF (D1A7) XP® Rabbit mAb detects endogenous levels of total CTCF protein. Species Reactivity: Human, Rat, Monkey".
- Anti-MBP (goat; Santa Cruz Biotechnology, sc-13914): validated by the manufacturer " MBP (C-16) is recommended for detection of MBP of mouse, rat and human origin by Western Blotting (starting dilution 1:200, dilution range 1:100-1:1000), immunofluorescence (starting dilution 1:50, dilution range 1:50-1:500)".
- Anti-MPZ (rabbit; Abcam, ab31851): validated by the manufacturer " Tested applications Suitable for: WB, IHC-FoFr, IHC-P, ICC/IF. Species reactivity Reacts with: Mouse, Rat, Human".
- Anti-EGR2 (rabbit, Santa Cruz Biotechnology, sc-20690): validated by the manufacturer "Egr-2 (H-220) is recommended for detection of Egr-2 of mouse, rat and human origin by Western Blotting (starting dilution 1:200, dilution range1:100-1:1000), immunofluorescence (starting dilution 1:50, dilution range 1:50-1:500)".
- Anti-EGR2/Krox20 (guinea pig; generated against a bacterially expressed and purified peptide corresponding to amino acids 28–166 of mouse Krox20 according to accession number NM_010118.3, validated on mouse sciatic nerve with and without Krox20 expression, 1:1000 dilution; PMID: 31142747)
- Anti-SUZ12 (rabbit, Cell Signaling Technology, 3737S): validated by the manufacturer "Application: Western Blotting, Immunoprecipitation. Specificity / Sensitivity: SUZ12 (D39F6) XP® Rabbit mAb detects endogenous levels of SUZ12 protein. Species Reactivity:Human, Mouse, Rat, Monkey".
- Anti-EED (rabbit, Millipore, 17-10034): validated by the manufacturer "Key Applications: Western Blotting. Specificity: Recognizes EED, Mr 62-70 kDa. Species Reactivity: Human, Mouse, Rat, Opossum, Chicken, Canine, Bovine".
- Anti-EZH2 (rabbit, Cell Signaling Technology, 5246P): validated by the manufacturer "Application: Western Blotting. Specificity / Sensitivity: Ezh2 (D2C9) XP® Rabbit mAb detects endogenous levels of total Ezh2 protein. This antibody does not cross-react with Ezh1 protein. Species Reactivity: Human, Mouse, Rat, Monkey".
- Anti-H3 (rabbit, Cell Signaling Technology, 4499S): validated by the manufacturer " Application: Western Blotting. Specificity / Sensitivity: Histone H3 (D1H2) XP® Rabbit mAb detects endogenous levels of total Histone H3 protein, including isoforms H3.1, H3.2, and H3.3. This antibody also detects the Histone H3 variant CENP-A. This antibody does not cross-react with other core histones. Species Reactivity: Human, Mouse, Rat, Monkey".
- Anti-H3K27me3 (rabbit, Cell Signaling Technology, 9733S): validated by the manufacturer "Application: Western Blotting, Chromatin IP, Chromatin IP-seq. Specificity / Sensitivity: Tri-Methyl-Histone H3 (Lys27) (C36B11) Rabbit mAb detects endogenous levels of

histone H3 only when tri-methylated on Lys27. The antibody does not cross-react with non-methylated, mono-methylated or di-methylated Lys27. In addition, the antibody does not cross-react with mono-methylated, di-methylated or tri-methylated histone H3 at Lys4, Lys9, Lys36 or Histone H4 at Lys20. Species Reactivity: Human, Mouse, Rat, Monkey".
- Anti-H3K27me2/3 (mouse, Active motif, 39536): validated by the manufacturer "Validated Applications: WB: 0.5 - 2 µg/ml dilution. Reactivity: Human, Wide Range Predicted".
- Anti-H3K36me3 (rabbit, Abcam, ab9050): validated by the manufacturer "Suitable for: ICC/IF, CHIPseq, WB, ChIP, ChIP/Chip, IHC-P. Reacts with: Mouse, Rat, Cow, Human".
- Anti-H3K4me1 (mouse, Active motif, 39635): validated by the manufacturer "Validated Applications: WB: 1:500 - 1:2,000 dilution. Reactivity: Human, Wide Range Predicted".
- Anti-H3K27ac (rabbit, Cell Signaling Technology, 4353S): validated by the manufacturer "Application: Western Blotting. Specificity / Sensitivity: Acetyl-Histone H3 (Lys27) Antibody detects endogenous levels of histone H3 when acetylated on Lys27. This antibody shows weak cross-reactivity with histone H3 acetylated on Lys9. This antibody does not cross-react with Histone H3 acetylated on lysines 14, 18 and 56. Species Reactivity: Human, Mouse, Rat, Monkey".
- Anti-GAPDH (mouse, Millipore, MAB374): validated by the manufacturer "Application: Anti-Glyceraldehyde-3-Phosphate Dehydrogenase Antibody, clone 6C5 is a well published and extensively characterized monoclonal antibody. Western blot: 1:100 to 1:300. Recognizes a 36kDa band of the reduced monomer. species reactivity: rabbit, feline, mouse, rat, fish, pig, canine, human".
- Anti-Flag (rabbit, 14793S or mouse, 8146S, Cell Signaling Technology): validated by the manufacturer " Application: Western Blotting, Immunoprecipitation. Specificity / Sensitivity: DYKDDDDK Tag (D6W5B) Rabbit mAb detects exogenously expressed DYKDDDDK proteins in cells. The antibody recognizes the DYKDDDDK peptide, which is the same epitope recognized by Sigma's Anti-FLAG® antibodies, fused to either the amino-terminus or carboxy-terminus of the target protein".
- Anti-HA-tag (Mouse, Cell Signaling Technology, 2367S): validated by the manufacturer " Application: Western Blotting. Specificity / Sensitivity: HA-Tag (6E2) Mouse mAb detects recombinant proteins containing the HA epitope tag. The antibody recognizes the HA-tag fused to either the amino or carboxy terminus of targeted proteins in transfected cells".
- Anti-CTCF (rabbit; Cell Signaling, #3418): validated by the manufacturer "Application: Immunofluorescence (Immunocytochemistry), Chromatin IP, Chromatin IP-seq. Specificity / Sensitivity: CTCF (D31H2) XP® Rabbit mAb detects endogenous levels of total CTCF protein. Species Reactivity: Human, Mouse, Rat, Monkey".
- Anti-SOX10 (goat, Santa Cruz Biotechnology, sc-17342): validated by the manufacturer " Sox-10 (N-20) is recommended for detection of Sox-10 of mouse, rat and human origin by immunofluorescence (starting dilution 1:50, dilution range 1:50-1:500)".
- Anti-SOX10 (rabbit, Abcam; ab155279): validated by the manufacturer "Suitable for: ICC/IF, IHC-FoFr, WB, Flow Cyt. Reacts with: Mouse, Rat, Human".
- Anti-Oct6 (goat; Santa Cruz Biotechnology, sc-11661): validated by the manufacturer "Oct-6 (C-20) is recommended for detection of Oct-6 of mouse, rat and human origin by immunofluorescence (starting dilution 1:50, dilution range 1:50-1:500)".
- Anti-SOX2 (goat; Santa Cruz Biotechnology, sc-17320): validated by the manufacturer "Sox-2 (Y-17) is recommended for detection of Sox-2 of mouse, rat and human origin by immunofluorescence (starting dilution 1:50, dilution range 1:50-1:500)".
- Anti-Ki67 (rabbit; Thermo Scientific, RM-9106-S0): Ki67 (Clone SP6) is excellent for staining and has been tested for use in immunofluorescence (Immunohistochemistry) on mouse species.
- Anti-BrdU (rat; Abcam, ab6326): validated by the manufacturer "Tested applications: Suitable for: ICC/IF, IHC-FoFr, IHC-P, IHC-Fr, Flow Cyt, IHC-FrFl".
- Anti-cleaved caspase 3 (rabbit; Cell Signaling, #9661): validated by the manufacturer "Application: Immunofluorescence (Immunocytochemistry). Specificity / Sensitivity: Cleaved Caspase-3 (Asp175) Antibody detects endogenous levels of the large fragment (17/19 kDa) of activated caspase-3 resulting from cleavage adjacent to Asp175. This antibody does not recognize full length caspase-3 or other cleaved caspases. Species Reactivity: Human, Mouse, Rat, Monkey".
- Anti-NF-M (Rabbit, Millipore, AB1987): validated by the manufacturer "Anti-Neurofilament M (145 kDa) Antibody, C-terminus detects level of Neurofilament M (145 kDa) & has been published & validated for use in IC, IH, IH(P) & WB. Species reactivity: reptile, human, pig, rat, bovine, mouse, rabbit, chicken, quail".
- Anti-H3K27Ac (Active motif, 39135): validated by the manufacturer "Validated Applications: ChIP: 3 µl per ChIP, ChIP-Seq: 5 µl each. Reactivity: Budding Yeast, Human, Wide Range Predicted".

Eukaryotic cell lines

Policy information about cell lines

Cell line source(s)	Rat Schwann cells from sciatic nerves of newborn rats (1–2 d old) were isolated as described in Methods.<br>HEK293T cell line was purchased from ATCC.
Authentication	Rat Schwann cells were authenticate by immunostaining for SOX10 and S100β, and >95% SC purity was achieved.<br>HEK293T cell line was authenticated by ATCC.
Mycoplasma contamination	All cell lines tested negative for mycoplasma contamination.
Commonly misidentified lines<br>(See ICLAC register)	No commonly misidentified cell lines were used in the study.

Animals and other organisms

Policy information about studies involving animals; ARRIVE guidelines recommended for reporting animal research

Laboratory animals	The mouse species used in this study, and a mixd C57Bl/6;129Sv strain was used.<br>The animal were housed in a vivarium with a 12-h light/dark cycle. Ambient temperature (22°C) and 30-70% humidity was maintained. No more than 4 adult mice were housed in the same cage per IACUC regulations at CCHMC.<br>Both male and female mice were used for the present study.

	For immunofluorescence of CTCF, SOX10 in SC nuclei, mice at P7, P14, and P62 were used (Figure 1)<br>For RT-qPCR of Ctcf, mice at P0.5, P7, P10, P21, P60 were used. (Figure 1)<br>For expression of CTCF, SOX10, MBP, Prx, Mpz, BrdU, Ki67, Cleaved-caspase 3, mice at P7 were used (Figure 3, 4)<br>For survival rate, mice from P0.5 to P80 were used (Figure 3)<br>For observation of sciatic nerve, mice at P13 were used (Figure 3)<br>For EM of sciatic nerve, mice at P1, P7 and 8 weeks were used (Figure 3)<br>For TAM injection of pups, mice at P0 were used (Figure 3)<br>For expression of Egr2, Oct6, Sox10, mice at P2, P7, P14, P21, P28 were used (Figure 4)<br>For injury experiment, mice from 6-8 weeks were used (Figure 5)<br>For RNA-seq of sciatic nerves, mice at P7 were used (Figure 6)<br>For TAM injection of adult mice, mice at about 6 week were used (Supplementary figure 1)
Wild animals	The study did not involve any samples collected from the wild animals.
Field-collected samples	The study did not involve any samples collected from the field.
Ethics oversight	All animal use and studies were approved by the Institutional Animal Care and Use Committee at Cincinnati Children's Hospital Medical Center.

Note that full information on the approval of the study protocol must also be provided in the manuscript.

ChIP-seq

Data deposition

☒ Confirm that both raw and final processed data have been deposited in a public database such as GEO.

☒ Confirm that you have deposited or provided access to graph files (e.g. BED files) for the called peaks.

Data access links<br>*May remain private before publication.*	Generated by this study:<br>https://www.ncbi.nlm.nih.gov/geo/query/acc.cgi?acc=GSE138117<br>Other datasets:<br>https://www.ncbi.nlm.nih.gov/geo/query/acc.cgi?acc=GSE84265<br>https://www.ncbi.nlm.nih.gov/geo/query/acc.cgi?acc=GSE93161
Files in database submission	FOR RAW FILES<br>CTCF_diff_chipseq.fastq.gz<br>CTCF_pro_chipseq.fastq.gz<br>run1879_lane1_read1_indexA003=Jincheng-rSC-Scramble-H3K27ac.fastq.gz<br>run1879_lane1_read1_indexA006=Jincheng-rSC-siCTCF-H3K27ac.fastq<br><br>FOR PROCESSED FILES<br>CTCF_diff_chipseq.wig<br>CTCF_pro_chipseq.wig<br>Jincheng-rSC-Scramble-H3K27ac.wig_temp.wig.gz<br>Jincheng-rSC-siCTCF-H3K27ac.wig_temp.wig.gz
Genome browser session<br>(e.g. UCSC)	No longer applicable

Methodology

Replicates	One replicate for CTCF ChIP-seq in proliferative and differentiated rat Schwann cells<br>One replicate for H3K27ac ChIP-seq in siControl and siCtcf rat Schwann cells
Sequencing depth	Pro_CTCF, total reads, 5567648, uniquely mapped-reads, 5455434;<br>diff_CTCF, total reads, 18976205, uniquely mapped-reads, 18052061;<br>siControl_H3K27ac, total reads, 22245258, uniquely mapped-reads, 22245258;<br>siCtcf_H3K27ac, total reads, 23030389, uniquely mapped-reads, 23030389
Antibodies	CTCF (rabbit; Cell Signaling, #3418)<br>H3K27Ac (Active motif, 39135)
Peak calling parameters	ChIP-seq peak calling was performed as previously described using MACS (Modelbased Analysis of ChIP-seq) (http://liulab.dfci.harvard.edu/MACS)<br>with default parameters. windows size 300, p-value 1e-5, MFOLD enrichment 32,<br>bowtie 2 with default settings on rn5
Data quality	To ensure that our data were of high quality and reproducibility, we called peaks with enrichment ≥ 10-fold over control (p ≤ 10-9) and compared the peak sets using the ENCODE overlap rules. These identified primary regions were further filtered using the following criteria, to define a more stringent protein–DNA interactome: (1) the p-value cutoff was set to ≤ 10−9; (2) an enrichment of

6-fold and peak height > 5. We also used the FastQC pipeline to measure data quality of ATAC-seq and ChIP-seq data, and all the ATAC-seq and ChIP-seq sequencing data past the quality control criteria (e.g. Basic Statistics: pass; Per base sequence quality: pass; Sequences flagged with poor quality: none) as indicated in Supplementary Data 4.

Software	The genome-wide distribution of protein binding regions was determined by HOMER (http://homer.salk.edu/homer/index.html) in reference to Ensembl RGSC3.4.61 release. For all ChIP-seq data sets, WIG files were generated with MACS, which were subsequently visualized using Mochiview v1.46. ChIP-seq heatmaps were ordered by strength of binding. The heatmaps were drawn using the Heatmap tools provided by Cistrome (http://cistrome.org/ap).
